# Apigenin Suppresses Bladder Cancer via the SIRT6-NCOA2-PPARα Axis

**DOI:** 10.7150/ijbs.128177

**Published:** 2026-02-26

**Authors:** Ying Liu, Zhen-Duo Shi, Yun-Fei Wei, Si-Yuan Jiang, Harsh Patel, Qian-Zi Liu, Yang Dong, Yi-Fang Liu, Lin Hao, Shan Gao, Dong-Hua Yang, Qiang Li, Cong-Hui Han

**Affiliations:** 1 School of Life Sciences, Jiangsu Normal University, Xuzhou, China.; 2 Jiangsu Provincial Engineering Research Center of Cancer Cell Therapy and Translational Medicine, Xuzhou Central Hospital, Southeast University, Xuzhou, China.; 3 Department of Urology, Xuzhou Central Hospital, Southeast University, Xuzhou, China.; 4 Department of Urology, Affiliated Hospital of Nanjing University of Chinese Medicine, Jiangsu Province Hospital of Chinese Medicine, Nanjing, China.; 5 Department of Urology, Jiangsu Provincial Hospital of Chinese Medicine - Jiangbei Branch, Nanjing, China.; 6 Zhongda Hospital, Medical School, Advanced Institute for Life and Health, Southeast University, Nanjing, China.; 7 Department of Pharmaceutical Sciences, College of Pharmacy and Health Sciences, St. John's University, Queens, New York, USA.; 8 Department of Urology, Kuitun Hospital of Ili Kazakh Autonomous Prefecture, Kuitun, China.; 9 Zhongda Hospital, School of Life Sciences and Technology, Advanced Institute for Life and Health, Southeast University, Nanjing, China.; 10 New York College of Traditional Chines Medicine, Mineola, New York, USA.; 11 Xuzhou Institute of Agricultural Sciences in Jiangsu Xuhuai District, Key Laboratory of Biology and Genetic Breeding of Sweetpotato, Ministry of Agriculture and Rural Affairs, Sweetpotato Research Center, Xuzhou, China.

**Keywords:** bladder cancer, apigenin, SIRT6, NCOA2, metabolic reprogramming

## Abstract

Protein acetylation is increasingly recognized as a key regulator of tumor progression, yet natural compounds capable of modulating this modification remain poorly defined. Apigenin, a dietary flavonoid suppresses bladder cancer progression based on *in vitro* functional assays and dynamic xenograft models. Mechanistically, we applied an integrated multi-omics approach to unravel that apigenin enhances SIRT6-mediated deacetylation of Nuclear Receptor Coactivator 2 (NCOA2), leading to site-specific deacetylation of NCOA2 at lysine 780 and 785. This modification potentiates PPARα transcriptional activity, reprograms cellular energy metabolism, and disrupts mitochondrial membrane potential. Clinically, reduced SIRT6 expression coupled with elevated NCOA2 and mitochondrial/β-oxidation markers correlates with metastatic progression in bladder cancer. Together, these findings identify a previously unrecognized SIRT6-NCOA2-PPARα signaling axis as a metabolic vulnerability in bladder cancer.

## Introduction

Bladder cancer is the most prevalent malignancy of the urinary tract^1^-^4^, yet current surgical and intravesical therapies provide limited long-term benefit, with frequent recurrence and significant complications^5^-^8^. Epigenetic regulation, particularly protein acetylation and deacetylation, has emerged as a critical driver of gene expression and cellular processes in bladder cancer^9^, ^10^. However, the molecular mechanisms underlying these modifications and their therapeutic modulation remain largely unclear.

Emerging evidence indicates that protein acetylation is highly sensitive to cellular metabolic status and can be influenced by dietary factors and bioactive nutrients. In particular, several classes of dietary polyphenols and flavonoids have been shown to modulate epigenetic enzymes, including histone acetyltransferases and deacetylases, thereby reshaping transcriptional programs and cellular metabolism^9^, ^11^.

Apigenin (4',5,7-trihydroxyflavone)^12^, ^13^, a dietary flavonoid abundant in parsley, celery, and chamomile, exhibits broad-spectrum anti-tumor activity in colon, breast, prostate, and lung cancers by regulating apoptosis, cell cycle arrest, oxidative stress, and inflammation^14^-^17^. In bladder cancer, apigenin suppresses proliferation, induces G2/M phase arrest, and promotes apoptosis through caspase activation and modulation of Bcl-2 family protein via reactive oxygen species (ROS) and glutathione pathways^14^, ^18^, ^19^. More recently, vascular endothelial growth factor-β (VEGF-β) was identified as a direct target of apigenin in recurrent bladder cancer, linking its activity to the suppression of fibroblast-driven tumor progression^20^. Intriguingly, metabolic stress has been shown to alter intracellular acetyl-CoA pools, thereby influencing global protein acetylation^11^, suggesting that apigenin-mediated oxidative signaling may intersect with acetylation remodeling. To date, most studies on apigenin have focused on phenotypic outcomes such as ROS accumulation, mitochondrial dysfunction, and caspase activation^14^, ^18^, ^21^, while the impact on post-translational modifications and epigenetic remodeling is largely unknown. In particular, whether apigenin reshapes global protein acetylation to mediate its anti-tumor effects has not been addressed.

## Materials and Methods

### Human Bladder Tumor Samples

A retrospective analysis was performed on patients who underwent partial cystectomy or radical cystectomy due to bladder tumors at the Department of Urology, Xuzhou Central Hospital, between January 2022 and June 2024. All patients were diagnosed with urothelial carcinoma by postoperative pathology. Based on the surgical procedures and pathological findings, the tumor samples were classified into four groups: para-carcinoma tissues, primary bladder cancer tissues, recurrent bladder cancer tissues, and metastatic bladder cancer tissues. Written informed consent was obtained from all participants prior to tissue collection. The clinical characteristics of the patients are provided in Supplementary Table S1 and S2.

### Ethics Approval

All animal experiments were approved by the Animal Care and Treatment Committee of Jiangsu Normal University (JSNU-IACUC-2024067).

The study involving human tumor samples was approved by the Ethical Committee of Xuzhou Central Hospital (EC. XZXY-LI-20200708-024) and was conducted in accordance with established ethical guidelines.

### Cell Culture and Reagents

T24 cells (RRID: TCHu 55, Shanghai, China) and J82 cells (RRID: TCHu218, Shanghai, China) were obtained from the Institute of Cell Biology, Chinese Academy of Sciences, and cultured in RPMI-1640 medium (Gibco, USA, 11875093) supplemented with 10% fetal bovine serum (Gibco, USA, 1009-148) and 1% penicillin/streptomycin (Gibco, USA, 15140122). Cells were maintained at 37°C in a humidified atmosphere of 5% CO₂ and 95% air. Apigenin was purchased from Sigma-Aldrich (USA, 10798). A 100 mM stock solution was prepared in dimethyl sulfoxide (DMSO), aliquoted, and stored at -20°C protected from light. For all experiments, apigenin working solutions were freshly prepared by diluting the stock solution into culture medium immediately before use, and light exposure was minimized during preparation and handling. The final concentration of DMSO in cell culture medium did not exceed 0.1%.

### Bulk RNA Sequencing and Analysis

Total RNA was extracted using TRIzol® Reagent (Magen, China, R4801-02) following the manufacturer's protocol. RNA purity was evaluated by A260/A280 absorbance using a NanoDrop ND-2000 spectrophotometer (Thermo Scientific, USA), and RNA integrity (RIN > 7.0) was assessed using an Agilent Bioanalyzer 4150 system (Agilent Technologies, USA). Qualified samples were used for library construction.

Paired-end RNA-seq libraries were prepared using the VAHTS Universal V6 RNA-seq Library Prep Kit for Illumina (Vazyme, China, NR604-01). Poly(A)+ RNA was isolated from 1 μg total RNA using oligo(dT) beads, fragmented in First Strand Synthesis Buffer (ABclonal, China), and reverse transcribed with random hexamers and RNase H. Second-strand synthesis was performed with DNA Polymerase I, RNase H, dNTPs, and buffer. Double-stranded cDNA was ligated with adaptors and amplified by PCR. PCR products were purified using AMPure XP beads (Beckman Coulter, USA) and assessed with a Bioanalyzer 4150. Libraries were sequenced on an Illumina NovaSeq 6000 platform, generating 150 bp paired-end reads.

Quality control was conducted using in-house Perl scripts to remove adaptors and filter reads with >60% low-quality bases (Q ≤ 25) or >5% ambiguous bases (N). Clean reads were aligned to the reference genome using HISAT2^22^. Gene expression levels were calculated as FPKM using FeatureCounts^23^, based on read counts and gene lengths. Differential expression was analyzed using DESeq2^24^; genes with |log2FC| > 1 and Padj < 0.05 were considered significantly differentially expressed.

GO and KEGG enrichment analyses were performed using the dplyr package; functions/pathways with *p* < 0.05 were considered significantly enriched. Transcription factors (TFs) among DEGs were identified using AnimalTFDB 3.0 or, for unlisted species, via Pfam domain annotation (InterProScan) and the DBD database. Protein-protein interaction (PPI) networks were constructed using STRING; for unlisted species, homology-based mapping was done with BLASTx.

Alternative splicing (AS) events were identified using rMATS^25^, including SE, A5SS, A3SS, MXE, and RI types.

### Proteomics Analysis

Protein extraction was performed in SDC (sodium deoxycholate) buffer (5% SDC, 100 mM Tris-HCl, pH 8.5). Lysates were sonicated, boiled for 15 minutes, and centrifuged at 14,000g for 40 minutes. Protein concentration was measured using the BCA Protein Assay Kit (Beyotime, China, P0010). Proteins (15 μg/sample) were mixed with 5x loading buffer, boiled for 5 minutes, and separated on 4%-20% SDS-PAGE gels. Gels were stained with Coomassie Blue R-250.

For digestion, proteins were reduced with DTT (dithiothreitol), alkylated with IAA (iodoacetamide), and digested with trypsin (Promega, USA, V5111) overnight at 37°C. Peptides were desalted on MCX columns (OmicSolution, China, OS-MCX-1ML), vacuum-concentrated, and reconstituted in 0.1% formic acid. iRT peptides were added for retention time calibration.

Peptides were analyzed on an Orbitrap Astral mass spectrometer (Thermo Scientific, USA) coupled with Vanquish Neo UHPLC (Thermo Scientific, USA) in DIA mode. DIA-NN (v1.8.1) 26 was used for data analysis with trypsin specificity, one missed cleavage, fixed carbamidomethyl (C), and dynamic oxidation (M) and acetylation (Protein N-term). Protein identification required FDR ≤ 1%.

After preprocessing, protein expression data were Z-score standardized. Soft clustering was performed using the R package MFuzz 27, 28, with the fuzziness parameter (m) adjusted based on clustering flexibility. The number of clusters (6) was determined by minimizing within-cluster variation and considering the actual data trends. Each protein was assigned a membership score, and based on the maximum membership score, proteins were assigned to the main cluster. Cluster mean expression trends were plotted, and representative proteins were selected for downstream functional annotation.

Protein domain composition was analyzed using InterProScan^29^, which integrates databases like Pfam. Protein sequences were uploaded for automated annotation, and significant domains were selected for subsequent structural and functional inference.

Differential protein interactions were explored using the STRING database (v12.0), with a confidence threshold set to construct the PPI network. Network topology was analyzed to identify key node proteins.

### Establishment of CDX and PDX Models

CDX models: T24 cells were suspended in phosphate-buffered saline (PBS): Matrigel (1:1) at 1 × 10⁸ cells/mL. 100 μL was subcutaneously injected into male BALB/c nude mice. Once tumors reached 50-100 mm³, mice were randomized into groups (n = 6) and treated by oral gavage with vehicle (0.5% CMC-Na) or apigenin (Sigma-Aldrich, USA, 10798) at 150 or 300 mg/kg daily for 14 days. Tumor volume = (Length (L) × Width (W)²)/2. Mice were monitored for weight and general condition.

PDX models: PDX models were established by implanting ~50 mm³ bladder tumor fragments from patients into male NSG mice^30^. Upon reaching 50-100 mm³, mice received the same apigenin treatments and assessments as CDX models.

### Measurement of Serum Biochemical Parameters

Serum levels of alanine aminotransferase (ALT), aspartate aminotransferase (AST), blood urea nitrogen (BUN), and creatinine were determined using microplate-based colorimetric assay kits (Nanjing Jiancheng Bioengineering Institute, Nanjing, China) according to the manufacturer's instructions.

ALT (Vital health biotech, Nanjing China, C009-2-1) and AST (Vital health biotech, Nanjing China, C010-2-1) activities were measured based on the reaction between enzymatically generated pyruvate or oxaloacetate and 2,4-dinitrophenylhydrazine, forming a brown-colored hydrazone. The absorbance was read at 510 nm using a microplate reader. Enzyme activities were calculated by referencing a standard curve.

BUN concentration was assessed using a urease-glutamate dehydrogenase (GLDH)-based method (Vital health biotech, Nanjing China, C013-3-1). Urea was enzymatically converted to ammonium, which reacted with NADPH and α-ketoglutarate in the presence of GLDH. The decrease in absorbance at 340 nm was recorded, and urea levels were calculated accordingly.

Serum creatinine levels were measured using a creatininase-creatinase-aminoacid oxidase-peroxidase coupled assay (Vital health biotech, Nanjing China, C011-2-1). Creatinine was ultimately converted to hydrogen peroxide, which reacted with chromogenic substrates to produce a purple product. The change in absorbance at 546 nm was used for quantification.

All measurements were performed in triplicate (n = 3). Data were expressed as mean ± SD.

### Popliteal Lymph Node Metastasis Model

Male BALB/c nude mice aged 3-5 weeks (20-22 g) were purchased from GemPharmatech Co., Ltd and maintained in a specific pathogen-free (SPF) barrier system (License No.: SYXK (Su) 2018-0008). The mice underwent testing one week after acclimatizing. Housing conditions included individual ventilation systems, maintaining a temperature of 22-24 °C, humidity between 40 and 60%, and a 12/12 h light/dark cycle. During the experiment, the mice had free access to standard rodent diet and tap water. At the same time, we monitored the mice's food intake and weight changes to observe their health status. A total of 3 × 10⁶ transfected T24 cells (NCOA2, NCOA2 K780R/K785R, and NCOA2 K780Q/K785Q) in 50 μL PBS were injected into the paw pads of male BALB/c nude mice. After 6 weeks, metastasis was assessed via bioluminescence imaging. Mice were intraperitoneally injected with D-fluorescent potassium salt (10 μL/g body weight, 150 μg/mL; Meilunbio, China, MB1834), and anesthesia was maintained using isoflurane (RuiPu Bio, China). Imaging was performed with a bioluminescence detection system (LB983 NightOWL II, Berthold Technologies, Germany). Tumor volume = (L × W²)/2. Mice were monitored for weight and general condition^31^.

### Cellular Thermal Shift Assay (CETSA)

After washing and centrifuging the cells, collect the cells using PBS containing protease inhibitors and phosphatase inhibitors. The cells were snap-frozen with liquid nitrogen and then thawed at room temperature. This process was repeated 3 times before centrifuging at 15,000g to obtain cell proteins. Divide the cell proteins into two portions, and add equal volumes of apigenin and DMSO to each portion and incubate for 2 hours. Repack the samples, and heat each sample at a specified temperature of 40 to 60 °C for 3 minutes. After cooling and centrifuging, the supernatants collected were ready for Western blot analysis^32^.

### Immunohistochemistry (IHC)

Tumors were fixed in 4% paraformaldehyde, embedded in paraffin, sectioned at 5 μm, and subjected to antigen retrieval in citrate buffer. Endogenous peroxidase was blocked with 3% H₂O₂. Sections were blocked with 10% goat serum and incubated overnight at 4 °C with primary antibodies diluted in PBS: CPT1A (CST, USA, 12252), FASN (CST, USA, 3180). HRP-conjugated anti-rabbit secondary antibody (Abcam, UK, ab205718) was applied for 1 h at 37°C. Staining was visualized with DAB (Maxim Biotechnology, China, DAB4033). Images were captured with an Olympus BX41 microscope.

### Reactive Oxygen Species (ROS) Detection

T24 cells were treated with 100 μM apigenin alone for 4h, 8h, 12h or 24h; or treated with 100 μM apigenin and 5 μM NXT629 (MCE, China, HY-114263) for 24 h, stained with 10 μM DCFH-DA (Beyotime, China, S0033), and imaged by fluorescence microscopy (FITC filter, Olympus, Japan). For quantification, fluorescence was analyzed by flow cytometry (BD Biosciences, USA).

### Lipid Droplet Staining

For visualization of lipid droplets, T24 cells were treated with 40 μM apigenin for 24 hours. After treatment, cells were washed twice with PBS and then incubated with BODIPY (Beyotime, China, C2053S) at the concentration recommended by the manufacturer, for 30 minutes at 37 °C in the dark. Following incubation, excess dye was removed by washing the cells 3 times with PBS. Lipid droplets were then imaged using an inverted fluorescence microscope (Olympus, Japan) equipped with a FITC filter^33^.

### JC-1 Assay

To evaluate mitochondrial membrane potential (Δψm), T24 cells were treated with 100 μM apigenin alone for 4 h, 8 h, 12 h or 24 h; or treated with 100 μM apigenin and 5 μM NXT629 for 24 h. After treatment, cells were collected and incubated with JC-1 dye (Beyotime, China, C2006) according to the manufacturer's instructions at 37 °C for 30 minutes in the dark. Following incubation, cells were washed twice with PBS to remove excess dye and then analyzed using a flow cytometer (BD Biosciences, USA). The JC-1 monomer and aggregate fluorescence signals were detected in the FITC and PE channels, respectively. An increase in the proportion of JC-1 monomers relative to aggregates was interpreted as an indicator of decreased mitochondrial membrane potential. Data analysis was performed using FlowJo software (Tree Star, USA).

### MitoTracker Staining

To visualize mitochondria, T24 cells were treated with 100 μM apigenin alone for 4 h, 8 h, 12 h or 24 h; or treated with 100 μM apigenin and 5 μM NXT629 for 24 h. Following treatment, cells were washed twice with PBS and incubated with Mito-Tracker Green (Beyotime, China, C1048) at 100 nM for 30 minutes at 37 °C in the dark. After incubation, cells were washed 3 times with PBS to remove unbound dye. Fluorescence images were captured using an inverted fluorescence microscope (Olympus, Japan) equipped with a FITC filter. Quantification of mitochondrial fluorescence intensity was performed using ImageJ software (NIH, Bethesda, MD, USA).

### Western Blot Analysis

T24 cells were treated with 100 μM apigenin or 5 μM NXT629 for 24 h. Cells were lysed in RIPA buffer (Beyotime, China, P0013B) with protease (P1005) and phosphatase inhibitors (P1081). Supernatants were collected after 12,000 rpm centrifugation (30 minutes, 4 °C). Protein was quantified by BCA (Beyotime, China, P0009) and separated by SDS-PAGE. After transfer to PVDF, membranes were blocked and incubated with antibodies: CPT1A, FASN, ACC (CST, USA, 3662), GAPDH (Proteintech, China, 60004-1-Ig). Secondary antibodies: anti-mouse (CST, USA, 7076S), anti-rabbit (Jackson, USA, 111-035-003)^34^. Detection used Potent ECL kit (MultiSciences, China, P1425) and GBOX system (LI-COR, USA). Band intensity was quantified using ImageJ software (NIH, USA) by measuring grayscale values, and normalized to GAPDH as the internal control.

### SIRT6-Mediated NCOA2 Deacetylation Assay

HEK293T cells were transiently transfected with plasmids encoding FLAG-tagged NCOA2. The cells were cultured and harvested 48 hours post-transfection for protein extraction. FLAG-tagged NCOA2 was purified from cell lysates using FLAG® M2 affinity gel (Sigma Aldrich) following the manufacturer's protocol. Briefly, cells were lysed in a buffer containing 50 mM Tris-HCl (pH 7.4), 150 mM NaCl, 1 mM EDTA, 1% NP-40, supplemented with a protease inhibitor cocktail. After incubation with FLAG affinity gel, the resin was washed and the protein was eluted with 3×FLAG peptide (150 µg/mL). The eluate was concentrated using Amicon Ultra-15 centrifugal filter units (50 kDa MWCO), and buffer exchange was performed to achieve a final protein concentration of ≥ 500 µg/mL. Recombinant human SIRT6 (S40-30EG, sinobilogical, Beijing, China) and FLAG-NCOA2 proteins were prepared in either HEPES-based buffer (25 mM HEPES, pH 8.0, 100 mM NaCl, 0.2 mM TCEP, 0.01% Tween-20, 0.1 mg/mL BSA, 1 mM NAD^+^, with optional 50 μM ZnCl_2_). Enzymatic reactions were initiated by adding apigenin (0, 0.1, 0.2, 0.4, 0.8, 1, 5, 10, 20 μM) or DMSO as a control and incubated at 37 °C for 2 hours. After the reaction, proteins were denatured by adding SDS-PAGE loading buffer and boiled for 10 minutes. Acetylation levels were assessed by Western blot analysis. The expression of FLAG-tagged NCOA2 and its acetylation status were analyzed by SDS-PAGE followed by Western blotting. The deacetylation levels of NCOA2 were quantified using densitometric analysis of the blots.

### Quantitative real-time PCR (qRT-PCR)

RNA was isolated via TRIzol (G8011, Adams life, Shanghai, China) following the manufacturer's instructions and then converted into complementary deoxyribonucleic acid (cDNA) via reverse transcriptase (11155ES60, Yeasen, Shanghai, China). Gene expression was assessed via the use of 2×SYBR Green Master Mix according to the manufacturer's instructions (A57115, Thermo Fisher, Shanghai, China). qRT-PCR data were analyzed via the 2-^ΔΔCT^ method, and Gapdh was used as the housekeeping gene. The sequence information is shown in Table S3.

### Cell Proliferation Assays by CCK-8

Cells were treated with apigenin or N-acetylcysteine (NAC). Cell viability was measured using CCK-8 (Beyotime, China, C0037). Absorbance at 450 nm was read using a BioTek microplate reader (USA).

### Wound Healing Assay

T24 cells were seeded into 24-well plates and allowed to grow to 90-100% confluency. A sterile 200 μL pipette tip was used to create a straight scratch (wound) across the cell monolayer. Detached cells were gently removed by washing with PBS. Cells were then cultured in serum-free medium and treated with apigenin at indicated concentrations. Images of the wound area were captured at 0 and 24 hours using an inverted microscope (Olympus, Japan). The migration distance was measured using ImageJ software (NIH, USA), and the wound closure percentage was calculated^35^.

### Colony Formation Assay

T24 cells were seeded into 6-well plates at a density of 600 cells per well in complete medium. After 24 h of adherence, cells were treated with apigenin (100 μM) for 24 h. The drug-containing medium was then removed, and cells were washed twice with PBS before replenishing with fresh drug-free medium. Cells were cultured undisturbed to allow colony formation, with the medium replaced every 3 days. After incubation, colonies were fixed with 4% paraformaldehyde (Beyotime, China, P0099) for 20 minutes and stained with 0.5% crystal violet (Sigma-Aldrich, USA, V5265) for 30 minutes. Excess stain was removed by extensive rinsing with distilled water, and plates were air-dried. Colonies (> 50 cells) were photographed quantified using ImageJ software (NIH, USA) with the particle analysis function.

### EdU Incorporation

EdU incorporation was evaluated with BeyoClick™ EdU-488 Kit (Beyotime, China, C0071S). After 24 h apigenin treatment (0-100 μM), cells were pulsed with 10 μM EdU for 2 h, fixed in 4% paraformaldehyde, permeabilized with 0.3% Triton X-100, and stained. Nuclei were counterstained with DAPI. Imaging was performed using fluorescence microscopy; EdU-positive cells were quantified using ImageJ^36^.

### Co-immunoprecipitation (Co-IP)

Cells were lysed in RIPA buffer with inhibitors. Lysates were incubated with Protein A+G magnetic beads (Beyotime, China, P2179M) pre-coupled with NCOA2 (CST, USA, 96687), FLAG (Proteintech, China, 66008-4-Ig), or SIRT6 (Abcam, UK, ab191385) antibodies. Normal IgG served as control. After incubation and washing, bound proteins were eluted, resolved by SDS-PAGE, and detected by Western blot.

### Luciferase Reporter Assay

T24 cells were transfected with pPPARα-TA-Luc (Beyotime, China, D2762) and pRL-SV40-N (internal control). After 24 hours, cells were treated with apigenin and assayed using Dual-Luciferase Reporter Assay Kit (Beyotime, China, RG027). Luminescence was measured using a microplate luminometer^37^.

### Lentiviral Transduction

Wild-type and mutant NCOA2 (K780R/K785R and K780Q/K785Q) coding sequences were subcloned into the GV747 lentiviral expression vector (GeneChem, China), which contains a CMV promoter, a multiple cloning site (MCS), a self-cleaving T2A peptide, and a puromycin resistance gene. All constructs were verified by Sanger sequencing.

Lentiviruses were generated and packaged by GeneChem (China). Viral supernatants were used to transduce T24 bladder cancer cells in the presence of 8 μg/mL polybrene (Sigma-Aldrich, USA). After 5-7 days of selection with 2 μg/mL puromycin, uninfected control cells were fully eliminated.

### Statistical Analysis

All experiments were conducted in at least three independent biological replicates. Quantitative data are expressed as mean ± standard deviation (SD). Statistical analyses were performed using GraphPad Prism 10.0 (GraphPad Software, USA) and R version 4.3.0 (R Foundation for Statistical Computing, Austria). For comparisons between two groups, an unpaired two-tailed Student's t-test was applied. For comparisons among more than two groups, one-way analysis of variance (ANOVA) followed by Tukey's post hoc test was used to assess statistical significance.

## Results

### Apigenin Suppresses Bladder Cancer Progression

**Figure 1:**
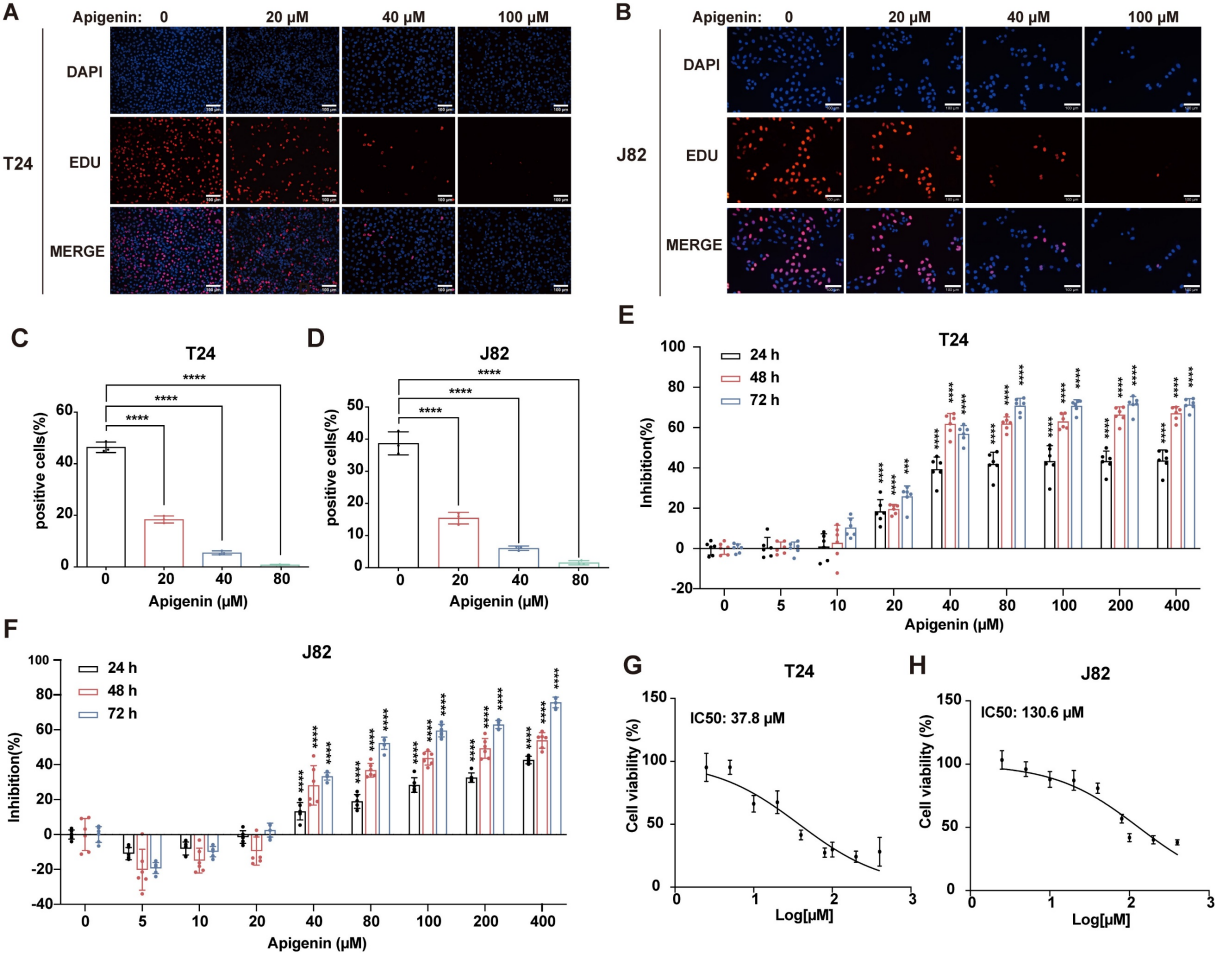
** Apigenin inhibits bladder cancer *in vitro*. (A-D)** T24 and J82 cells were treated with apigenin at concentrations of 0, 20, 40, and 100 μM for 24 h, and proliferation was evaluated using EdU incorporation. **(A-B)** Representative EdU fluorescence images. **(C-D)** Quantification of EdU-positive cells. **(E)** T24 cells were treated with increasing concentrations of apigenin (0, 5, 10, 20, 40, 80, 100, 200, and 400 μM) for 24 h, 48 h, and 72 h. Cell viability was assessed using the CCK-8 assay. **(F)** J82 cells were treated with increasing concentrations of apigenin (0, 5, 10, 20, 40, 80, 100, 200, and 400 μM) for 24 h, 48 h, and 72 h. Cell viability was assessed using the CCK-8 assay.** (G)** IC_50_ of apigenin in T24 cells at 48 h, as determined by the CCK-8 assay, was 37.8 μM. **(H)** IC_50_ of apigenin in J82 cells at 48 h, as determined by the CCK-8 assay, was 130.6 μM. Statistical comparisons were performed using one-way ANOVA followed by Tukey's post hoc test. Data are presented as mean ± SD. *P* values are indicated as follows: * *p* < 0.05, ** p < 0.01, *** p < 0.001, **** *p* < 0.0001; ns = not significant, compared to the control group.

**Figure 2:**
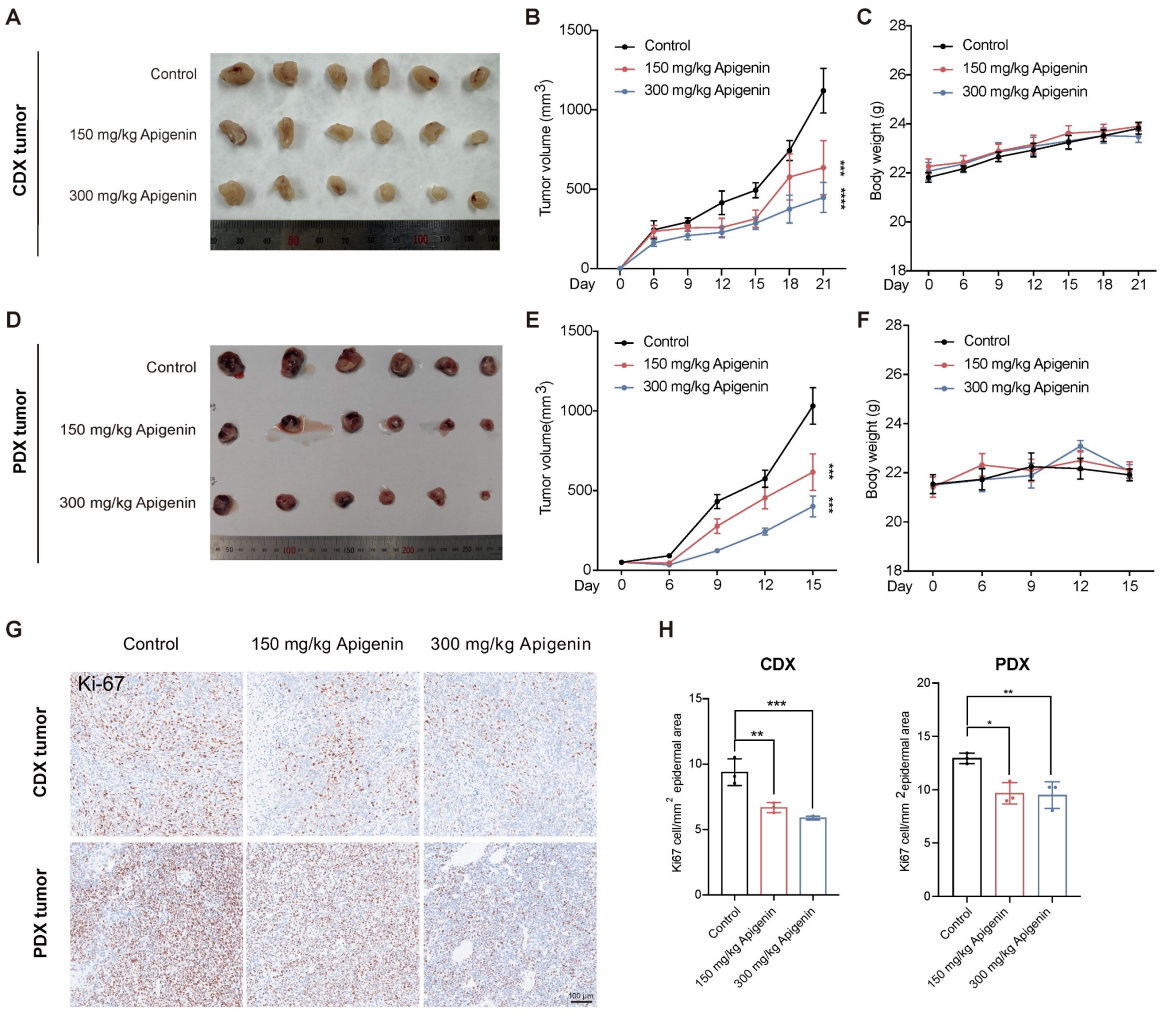
** Apigenin inhibits bladder cancer *in vivo*. (A-C)** Evaluation of tumor growth in the T24 cell-derived xenograft (CDX) mouse model (n=6) treatment with or without apigenin.** (A)** Representative images of excised tumors. **(B)** Tumor volume measurements recorded over the treatment period.** (C)** Body weight of CDX mice. **(D-F)** Evaluation of tumor growth in the patient-derived xenograft (PDX) mouse model (n=6). **(D)** Representative tumor images.** (E)** Tumor volume measurements over time. **(F)** Body weight of PDX mice. **(G)** Representative immunohistochemical images of Ki-67 staining in tumor tissues.** (H)** Quantification of Ki-67-positive cells across treatment groups (n=3). One-way ANOVA or two-way ANOVA followed by Tukey's post hoc test was used for group comparisons, as appropriate. Data are presented as mean ± SD. Statistical significance is indicated as: * p < 0.05, ** p < 0.01, *** p < 0.001, **** p < 0.0001; ns = not significant, compared to the control group.

To evaluate the antitumor activity of apigenin, we first evaluated its impact on T24 and J82 bladder cancer cell lines. EdU incorporation assays revealed a concentration-dependent reduction in DNA synthesis in both cell types, with T24 cells exhibiting greater sensitivity to apigenin treatment (Fig. 1A-D). Consistently, growth inhibition assays showed that T24 cells were more susceptible to apigenin-induced proliferation arrest compared to J82 cells (Fig. 1E-H). We next assessed the efficacy of apigenin *in vivo* using both cell-derived xenograft (CDX) model and patient-derived xenograft (PDX) models. Mice were treated with apigenin by daily oral gavage. After 14 days, apigenin significantly reduced tumor growth compared to controls (Fig. 2A and B), without affecting body weight or causing histological damage to major organs (Fig. 2C, Supplementary Fig. S1A and B). In the PDX model, tumor suppression was evident as early as 7 days after treatment starting (Fig. 2D-F). Immunohistochemical analysis of Ki-67 revealed that apigenin treatment significantly reduced the proportion of Ki67-positive cells in both CDX and PDX tumor models compared to control groups (Fig. 2G and H). Together, these results demonstrate that apigenin exerts potent anti-tumor activity against bladder cancer both* in vitro* and *in vivo*.

### Apigenin is Associated with Metabolic Reprogramming in Bladder Cancer

**Figure 3:**
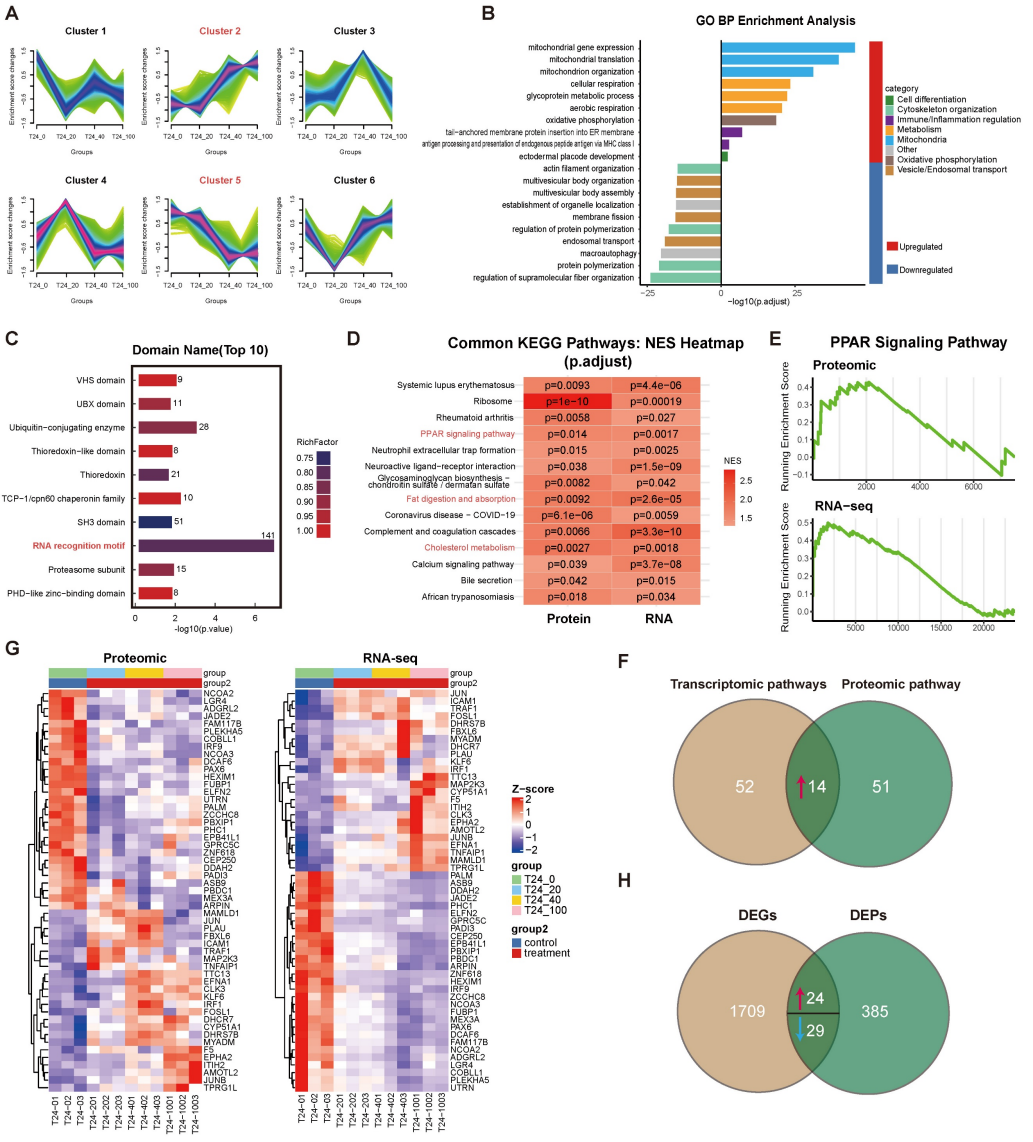
** Multi-omics profiling of dose-dependent apigenin responses in bladder cancer. (A)** Results of MFuzz soft clustering analysis on differentially expressed proteins from the proteomic data. A total of six clusters (Cluster 1-6) were identified. Each subplot shows the average normalized abundance change curves of proteins within the corresponding cluster under different treatment conditions (x-axis treatment doses ranging from control T24+0 to various drug concentrations of 20, 40, and 100 µM; y-axis Z-score normalized expression values). The shaded area represents the range of variation in protein expression within each cluster. **(B)** Gene Ontology (GO) biological process (BP) enrichment analysis of differentially expressed proteins. The bar chart displays the significantly enriched GO terms and their corresponding enrichment statistics. The x-axis represents the significance score, and the y-axis lists the names of the GO biological processes. **(C)** Domain enrichment analysis of differentially expressed proteins. The InterProScan tool was used to identify protein domains in the differentially expressed proteins, and the top 10 most significantly enriched domains were selected. The chart lists these domain names along with their enrichment statistics (x-axis enrichment factor or -Log10 *p*-values). **(D)** Heatmap of GSEA KEGG pathway enrichment shared by both proteomic and transcriptomic datasets. The rows represent KEGG pathway names, and the columns correspond to the proteomic and transcriptomic datasets. The color indicates the normalized enrichment score (NES) of each pathway in the corresponding dataset, with red representing positive enrichment (upregulated pathways) and blue representing negative enrichment (downregulated pathways). Color intensity reflects significance (only pathways with *p*<0.05 are shown). **(E)** GSEA enrichment curves for the PPAR signaling pathway in both proteomic and transcriptomic datasets. The upper graph shows the GSEA results for the proteomic data, and the lower graph shows the results for the transcriptomic data. The x-axis represents the rank of genes based on differential expression, and the y-axis shows the cumulative enrichment score. The green curve represents the enrichment of the PPAR pathway gene set within the sorted list. Both graphs show significant enrichment of PPAR pathway genes in the upregulated region of the curve in the drug-treated groups (with peak values towards the front, NES > 0, and *p*<0.05), indicating activation of PPAR downstream target genes at both the protein and mRNA levels. **(F)** Venn diagram comparing pathway enrichment between proteome and transcriptome. The left circle represents transcriptome-specific pathways (66 total), the right circle proteome-specific pathways (65 total), with 14 overlapping pathways significantly enriched in both datasets. **(G)** Heatmap of gene expression trends shared by both proteomic and transcriptomic datasets. The heatmap shows the expression levels of 53 differential genes in both proteomics (left) and transcriptomics (right). Rows represent genes/proteins, and columns represent biological replicates from different treatment groups. Color intensity ranges from blue (low expression) to red (high expression), indicating Z-score normalized expression values. **(H)** Venn diagram comparing DEGs and DEPs. The transcriptome identified 1,709 DEGs, the proteome identified 385 DEPs, and 24 genes were significantly changed at both transcript and protein levels.

To elucidate the molecular basis of apigenin's anti-tumor activity, we performed an integrated multi-omics analysis of T24 cells exposed to increasing apigenin concentrations. MFuzz clustering of proteomic profiles identified six distinct expression patterns (Fig. 3A). Among these, cluster 2 proteins were dose-dependently upregulated and enriched in mitochondrial and metabolic processes, suggesting activation of cellular energy metabolism. In contrast, cluster 5 proteins were progressively downregulated and associated with cytoskeletal remodeling, vesicular trafficking, and autophagy, indicating suppression of structural and transport pathways (Fig. 3B). Differential analysis (ANOVA) followed by domain enrichment analysis revealed significant enrichment of several protein domains including RNA recognition motif (RRM), SH3 domain, ubiquitin ligase domain, and proteasomal subunit, with RRMs being particularly prominent (Fig. 3C). These results suggest that apigenin modulates protein abundance and post-translational regulatory modules.

Gene Set Enrichment Analysis (GSEA) of transcriptomic and proteomic profiles, based on the Kyoto Encyclopedia of Genes and Genomes (KEGG) identified 14 significantly enriched pathways (Fig. 3D-F), including the Peroxisome proliferator-activated receptor (PPAR) signaling. Integrated analysis identified 53 concordantly regulated genes (24 upregulated, 29 downregulated) (Fig. 2G and H). The PPAR-centered gene set was associated with pronounced activation of oxidative phosphorylation, aerobic respiration and mitochondrial function, together with coordinated upregulation of lipid-handling pathways such as fat digestion and cholesterol homeostasis. These findings indicate that apigenin orchestrates a broad reprogramming of cellular energy metabolism and lipid homeostasis.

### Apigenin Induces Metabolic Reprogramming in Bladder Cancer

**Figure 4:**
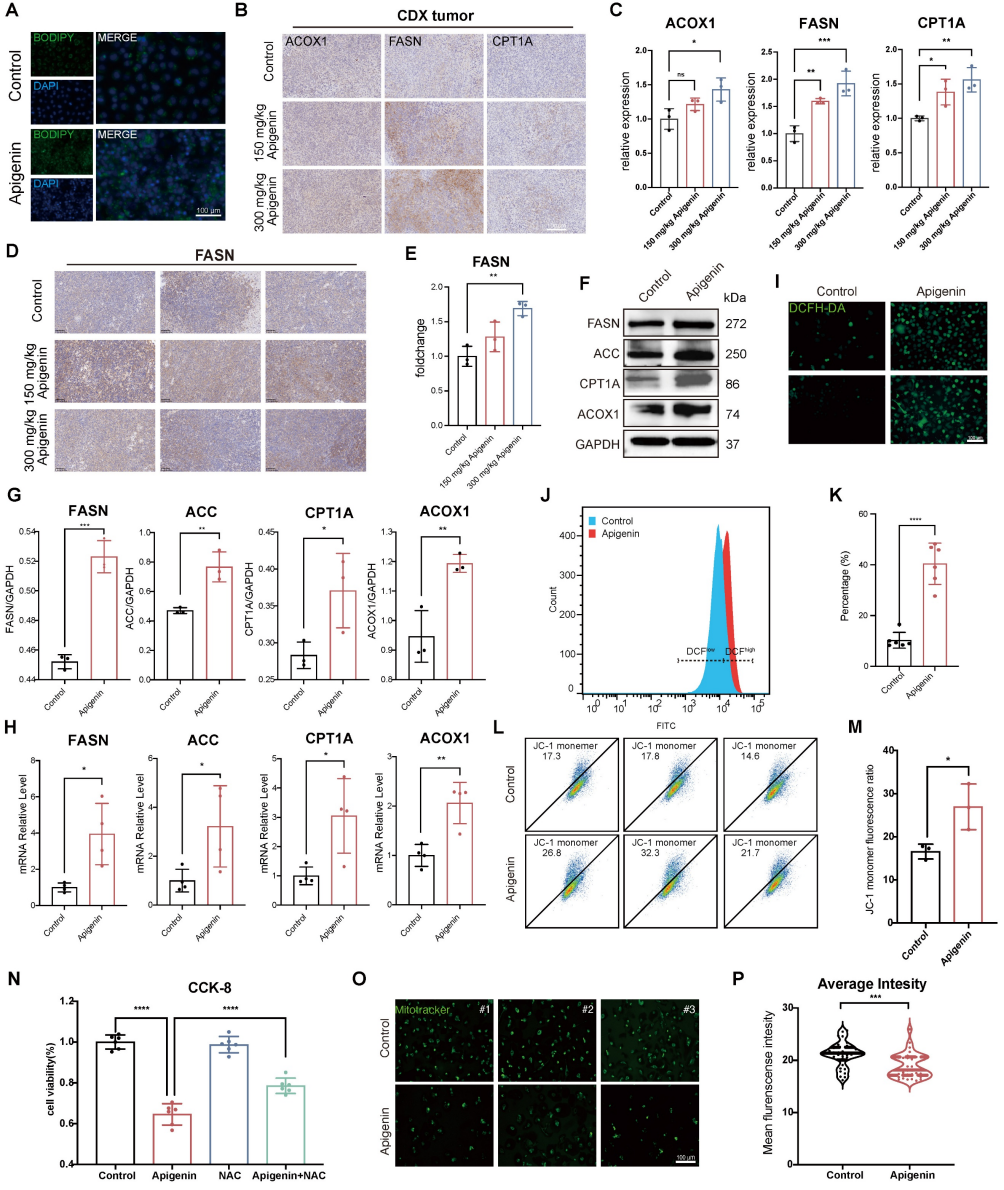
** Apigenin impairs mitochondria and promotes beta-oxidation. (A)** BODIPY staining shows increased accumulation of lipid droplets in T24 cells following 100 μM apigenin treatment. **(B)** Immunohistochemical (IHC) staining of tumor tissues from CDX mice treated with 300 mg/kg apigenin shows altered expression of CPT1A, FASN, and ACOX1. **(C)** Quantification of IHC staining intensity for Acyl-CoA oxidase 1 (ACOX1), Fatty Acid Synthase (FASN), and Carnitine Palmitoyltransferase 1A (CPT1A) (n=3). **(D)** Immunohistochemical staining of FASN in tumors derived from apigenin-treated PDX models. **(E)** Quantification of IHC staining intensity for FASN (n=3).** (F-H)** Treatment with 100 μM apigenin enhances the protein and mRNA expression of FASN, Acetyl-CoA Carboxylase (ACC), CPT1A, and ACOX1 (n=3). **(I-K)** Intracellular reactive oxygen species (ROS) levels were assessed using DCFH-DA. **(I)** Representative DCF fluorescence images; **(J-K)** Quantification of ROS levels by flow cytometry (n=6). **(L-M)** JC-1 staining indicates changes in mitochondrial membrane potential, with quantitative analysis performed by flow cytometry (n=3). **(N)** CCK-8 assay evaluates the viability of T24 cells co-treated with 100 μM apigenin and the 5 mM antioxidant N-acetylcysteine (NAC). **(O-P)** Mitochondrial content was assessed using MitoTracker staining.** (O)** Representative fluorescence images; **(P)** Quantification of MitoTracker fluorescence intensity. Statistical comparisons were performed using one-way ANOVA followed by Tukey's post hoc test. Data are presented as mean ± SD. *P* values are indicated as follows: * *p* < 0.05, ** p < 0.01, *** p < 0.001, **** *p* < 0.0001; ns = not significant, compared to the control group.

To further verify the impact on lipid metabolism, BODIPY staining revealed increased lipid droplet accumulation in apigenin-treated cells (Fig. 4A), indicating disrupted lipid homeostasis. Immunohistochemical (IHC) analysis of tumor specimens from CDX models demonstrated increased expression of Peroxisomal acyl-coenzyme A oxidase 1 (ACOX1), fatty acid synthase (FASN), and carnitine palmitoyltransferase 1A (CPT1A) upon apigenin exposure (Fig. 4B and C). Consistently, elevated FASN levels were also detected in PDX tumor tissues following apigenin administration (Fig. 4D and E). Analysis at the RNA and protein levels further confirmed significant upregulation of enzymes critical to fatty acid β-oxidation, including CPT1A, FASN, ACOX1, and acetyl-CoA carboxylase (ACC) (Fig. 4F-H). These transcriptional alterations reinforce a shift in lipid metabolic flux.

Beyond lipid metabolism, we found that Intracellular ROS were significantly elevated in T24 cells (Fig. 4I-K), concomitant with a marked reduction in mitochondrial membrane potential (MMP) (Fig. 4L-M), indicative of mitochondrial dysfunction. Importantly, co-treatment with the antioxidant N-acetylcysteine (NAC) significantly mitigated apigenin-induced cell death (Fig. 4N), supporting oxidative stress as a central mediator of mitochondrial impairment. To validate mitochondrial alterations, we performed Mitotracker staining, which consistently revealed reduced mitochondrial content after apigenin treatment (Fig. 4O and P), further substantiating mitochondrial loss as a consequence of redox and metabolic imbalance. In summary, our findings demonstrate that apigenin reprograms bladder cancer metabolism by enhancing fatty acid β-oxidation and lipid accumulation while impairing mitochondrial function, leading to oxidative stress and cellular damage.

### Apigenin promotes PPARα signaling via SIRT6-mediated NCOA2 deacetylation

**Figure 5:**
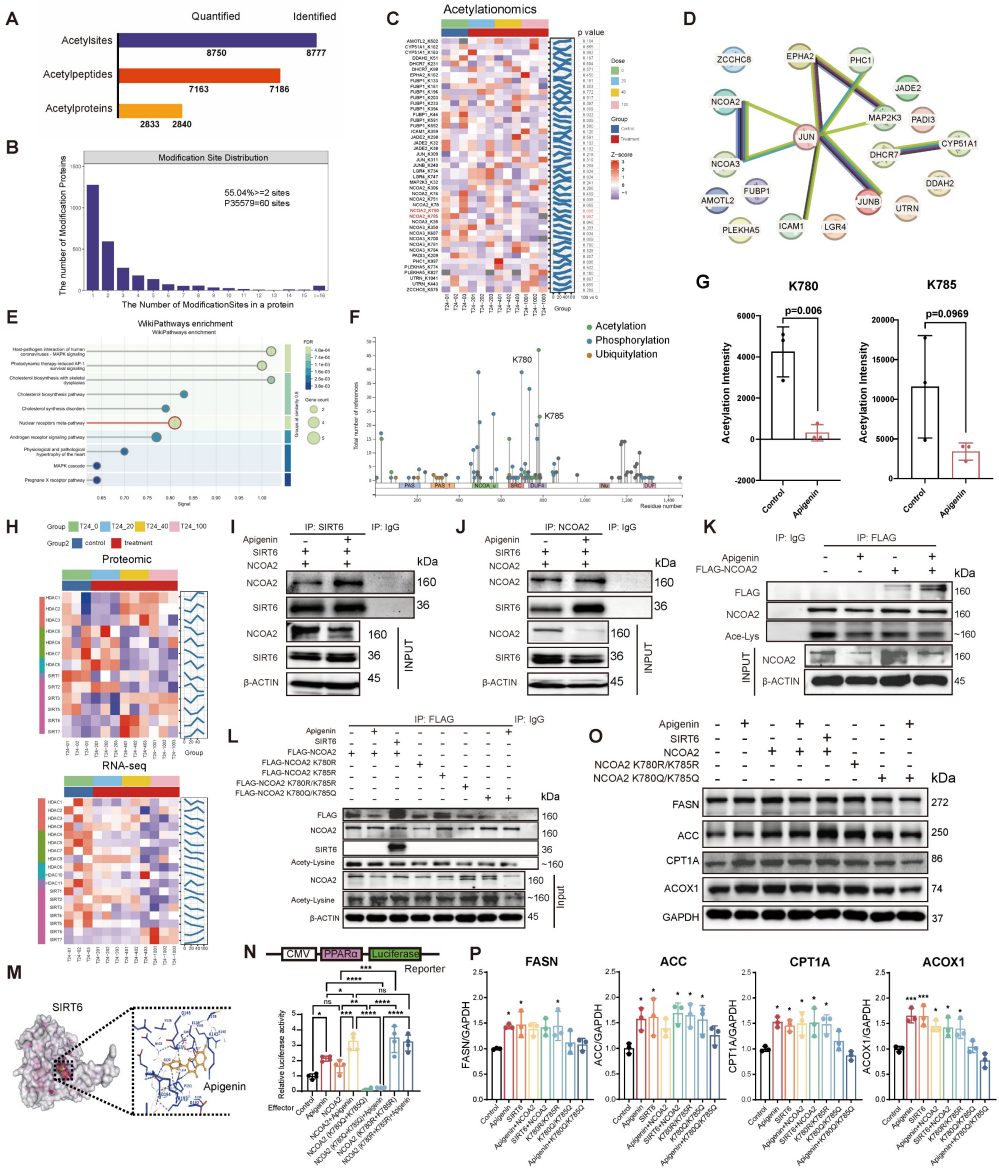
** Apigenin enhances SIRT6-NCOA2 interaction, promotes NCOA2 deacetylation, and is associated with PPARα signaling activation. (A)** Quantification summary of acetylated proteomics data. Paired bar graphs display identified and quantified acetylation sites, peptides, and proteins. **(B)** The number of modification sites in a protein. **(C)** Clustering heatmap of differential acetylation sites. Each row represents a lysine acetylation modification site (labeled as "protein name_K position"), and each column represents a sample (including the control group and various treatment doses). The right panel presents the p value for the comparison between the highest dose group (100) and the control group (0). The color reflects the relative acetylation levels of each site across the samples (Z-score) red indicates higher acetylation, while blue indicates lower acetylation. **(D)** Protein-Protein Interaction (PPI) Network of 19 genes. **(E)** WikiPathways enrichment analysis of differentially expressed proteins. Each circle represents an enriched pathway, with circle size proportional to the number of differentially expressed proteins in that pathway. The color corresponds to the statistical significance of the enrichment (warmer colors indicate more significant FDR values).** (F)** Schematic representation of post-translational modification sites in the NCOA2 protein. **(G)** Proteomic acetylation analysis reveals that apigenin treatment reduces acetylation of NCOA2 at lysine residues K780 and K785 (n=3). **(H)** Heatmap of deacetylase family protein expression. The upper graph shows proteomic data, and the lower graph shows transcriptomic data. Rows represent classical HDAC enzymes (classified as Class I, IIa, IIb, and IV) and Sirtuin family proteins (SIRT1-7), and columns correspond to different treatment group samples (T24+0 control and 20, 40, 100 µM treatment groups). The color represents the relative abundance of gene expression as a Z-score (red indicates higher expression compared to the control average, blue indicates lower expression). **(I-J)** Apigenin (100 μM) enhances the interaction between SIRT6 and NCOA2, as demonstrated by co-immunoprecipitation. **(K)** Acetylation levels of NCOA2 in T24 cells following apigenin treatment were detected using a pan-acetyl lysine antibody.** (L)** Pan-acetyl lysine antibody was used to assess acetylation levels of wild-type NCOA2 and its K780R, K785R, K780Q, K785Q single and double mutants following apigenin stimulation. **(M)** Molecular docking simulation of apigenin with SIRT6, showing a binding score of -7.6 kcal/mol. **(N)** Dual-luciferase reporter assays were conducted to evaluate PPARα transcriptional activity in T24 cells stably expressing NCOA2 K780R/K785R, K780Q/K785Q, or wild-type NCOA2 under apigenin treatment (n=4). **(O-P)** Apigenin enhances the expression of key enzymes involved in fatty acid β-oxidation, including FASN, ACC, CPT1A, and ACOX1, through deacetylation of NCOA2 at K780 and K785 sites (n=3). Statistical comparisons were performed using one-way ANOVA followed by Tukey's post hoc test. Data are presented as mean ± SD. *P* values are indicated as follows: * *p* < 0.05, ** p < 0.01, *** p < 0.001, **** *p* < 0.0001; ns = not significant, compared to the control group.

As lysine acetylation is a key regulator of metabolic enzymes and mitochondrial function, we next performed acetylome profiling to identify apigenin-induced modifications. The extensive lysine acetylation was observed, with most proteins harboring multiple modification sites (Fig. 5A and B). In total, 19 proteins exhibited significant acetylation changes across 45 acetylation sites upon apigenin treatment (Fig. 5C). To prioritize candidates linked to metabolic enzymes and mitochondrial function, we performed STRING (search tool for recurring instances of neighboring genes) analysis integrating transcriptomic and proteomic data. This revealed a JUN-centered protein interaction network (Fig. 5D), with nuclear receptor coactivators NCOA2 and NCOA3 forming a tightly associated sub-network. WikiPathways enrichment linked these proteins to the nuclear receptor meta-pathway (NCOA2/3) (Fig. 5E). Among these, NCOA2 stood out, as deacetylation at lysine residues K780/K785 has been previously implicated in metabolic control (Fig. 5F)^38^, ^39^. Consistently, apigenin treatment markedly reduced acetylation at these sites (Fig. 5G). Proteomic and transcriptomic analysis further revealed significant upregulation of deacetylases SIRT6 and SIRT7 after treatment (Fig. 5H). Given its known role in NCOA2 deacetylation, we focused on SIRT6. Apigenin enhancing the SIRT6-NCOA2 interaction (Fig. 5I and J), suggesting that enhanced NCOA2 deacetylation was primarily mediated by SIRT6. These effects paralleled enhanced fatty acid β-oxidation and reduced NCOA2 acetylation, aligning with prior reports that SIRT6 promotes fatty acid oxidation by deacetylating NCOA2^38^. This was accompanied by a global decrease in NCOA2 acetylation levels (Fig. 5K and L). To assess the potential direct regulation of SIRT6 by apigenin, molecular docking simulations and thermal shift assays were performed. The results indicated stable binding of apigenin to the catalytic pocket of SIRT6 (Fig. 5M, Supplementary Fig. S1C and D), although its effect on enzymatic turnover remains to be determined.

Functionally, NCOA2 deacetylation emerged as a critical upstream event for PPARα activation. Both apigenin treatment and deacetylation-mimicking NCOA2 mutants (K780R/K785R), but not acetylation-mimicking mutants (K780Q/K785Q), significantly enhanced PPARα transcriptional activity (Fig. 5N). Consistent with this observation, apigenin stimulation led to increased expression of key metabolic enzymes involved in fatty acid metabolism, including FASN, ACC, CPT1A, and ACOX1 (Fig. 5O and P). In conclusion, these findings demonstrate that apigenin correlates with increased PPARα activation through SIRT6-mediated deacetylation of NCOA2 at K780/K785, thereby promoting fatty acid metabolism and reprogramming energy homeostasis.

### Functional Role of NCOA2 Acetylation in Mitochondrial Dysfunction and Metastasis

**Figure 6:**
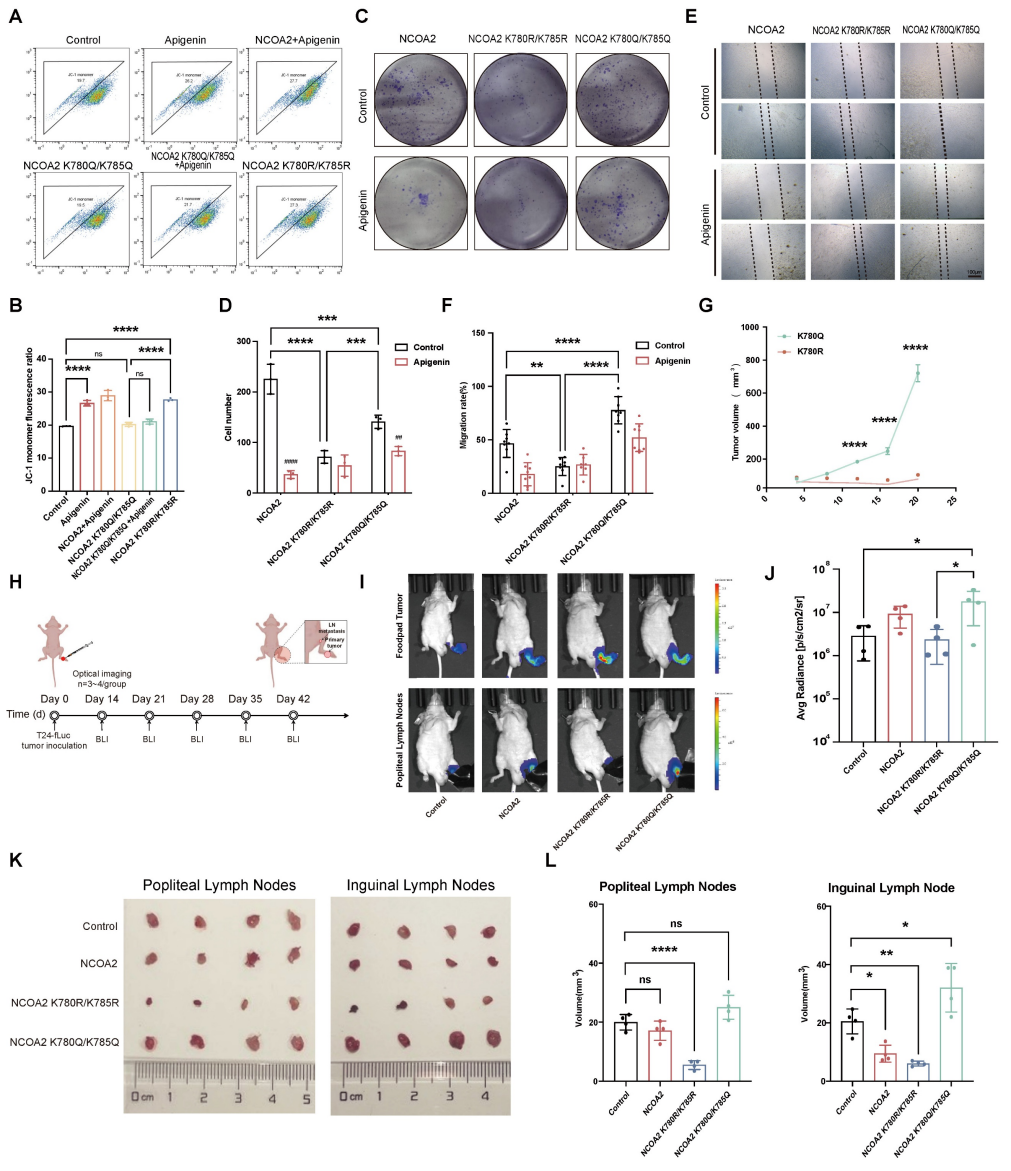
** NCOA2 Acetylation and its role in bladder cancer progression, metastasis. (A-B)** JC-1 staining analysis of mitochondrial membrane potential in T24 cells overexpressing wild-type NCOA2, the deacetylation-mimetic mutant (K780R/K785R), or the acetylation-mimetic mutant (K780Q/K785Q), with or without apigenin treatment. **(A)** Representative flow cytometry analysis of JC-1 fluorescence. **(B)** Quantitative analysis of red/green fluorescence intensity ratio (n=3). **(C-D)** Colony formation assay evaluating proliferation of T24 cells overexpressing wild-type or mutant NCOA2 under apigenin stimulation. **(C)** Representative colony images. **(D)** Quantification of colony numbers (n=3). **(E-F)** Wound healing assay assessing cell migration in T24 cells expressing wild-type or mutant NCOA2 following apigenin treatment. **(E)** Representative images at 0 h and 24 h.** (F)** Quantitative analysis of wound closure percentage (n=8).** (G)** Tumor growth evaluation in CDX mice implanted with NCOA2 K780R/K785R or K780Q/K785Q-mutated T24 cells (n=6).** (H-L)** *In vivo* lymph node metastasis model using T24 cells stably expressing wild-type NCOA2, K780R/K785R, or K780Q/K785Q.** (H)** Schematic diagram of the animal experiment protocol. **(I-J)** Representative images and quantitative analysis of tumor size in inguinal lymph nodes (n=4). **(K-L)** Representative images and quantitative analysis of tumor size in popliteal lymph nodes and Inguinal lymph nodes (n=4). All quantitative data are presented as mean ± SD from at least three independent experiments. Statistical comparisons were performed using one-way ANOVA followed by Tukey's post hoc test. Data are presented as mean ± SD. *P* values are indicated as follows: * *p* < 0.05, ** p < 0.01, *** p < 0.001, **** *p* < 0.0001; ns = not significant, compared to the control group.^ ##^ *p < 0.01,* ^###^* p < 0.001*, ^####^ *p* < 0.0001; ns = not significant, compared to the vehicle group.

Given mitochondrial activity is central to metabolic adaptation^40^, we generated acetylation- and deacetylation-mimicking NCOA2 mutants to examine the impact of acetylation on mitochondrial function. JC-1 staining assays demonstrated that the deacetylation-mimicking mutant markedly reduced mitochondrial membrane potential, whereas the acetylation-mimicking mutant prevented the apigenin-induced loss of mitochondrial membrane potential (Fig. 6A-B). Functionally, colony formation and wound-healing assays using T24 cells stably expressing these mutants revealed that the acetylation-mimicking mutant (K780Q/K785Q) enhanced proliferation and migration, while the deacetylation-mimicking mutant (K780R/K785R) preserved the suppressive effects of apigenin on both processes (Fig. 6C-F).

To assess the role of NCOA2 acetylation in metastasis *in vivo,* we established CDX models with T24 cells expressing wild-type NCOA2 or its mutants. Tumors derived from acetylation-mimicking cells exhibited robust growth, whereas deacetylation-mimicking cells failed to proliferate (Fig. 6G). In lymph node metastasis models (inguinal and popliteal) (Fig. 6H), acetylation-mimicking cells displayed markedly enhanced metastatic potential compared with wild-type or deacetylation-mimicking cells (Fig. 6I-L). Intriguingly, however, metastatic lesions derived from the acetylation-mimicking group were significantly smaller in volume than those from wild-type or deacetylation-mimicking cells, suggesting a potential uncoupling between metastatic dissemination and subsequent tumor expansion in the context of NCOA2 acetylation. These results suggest that SIRT6-mediated deacetylation of NCOA2 is associated with reduced metastatic potential in bladder cancer, and may contribute to the anti-metastatic effects of apigenin.

**Figure 7:**
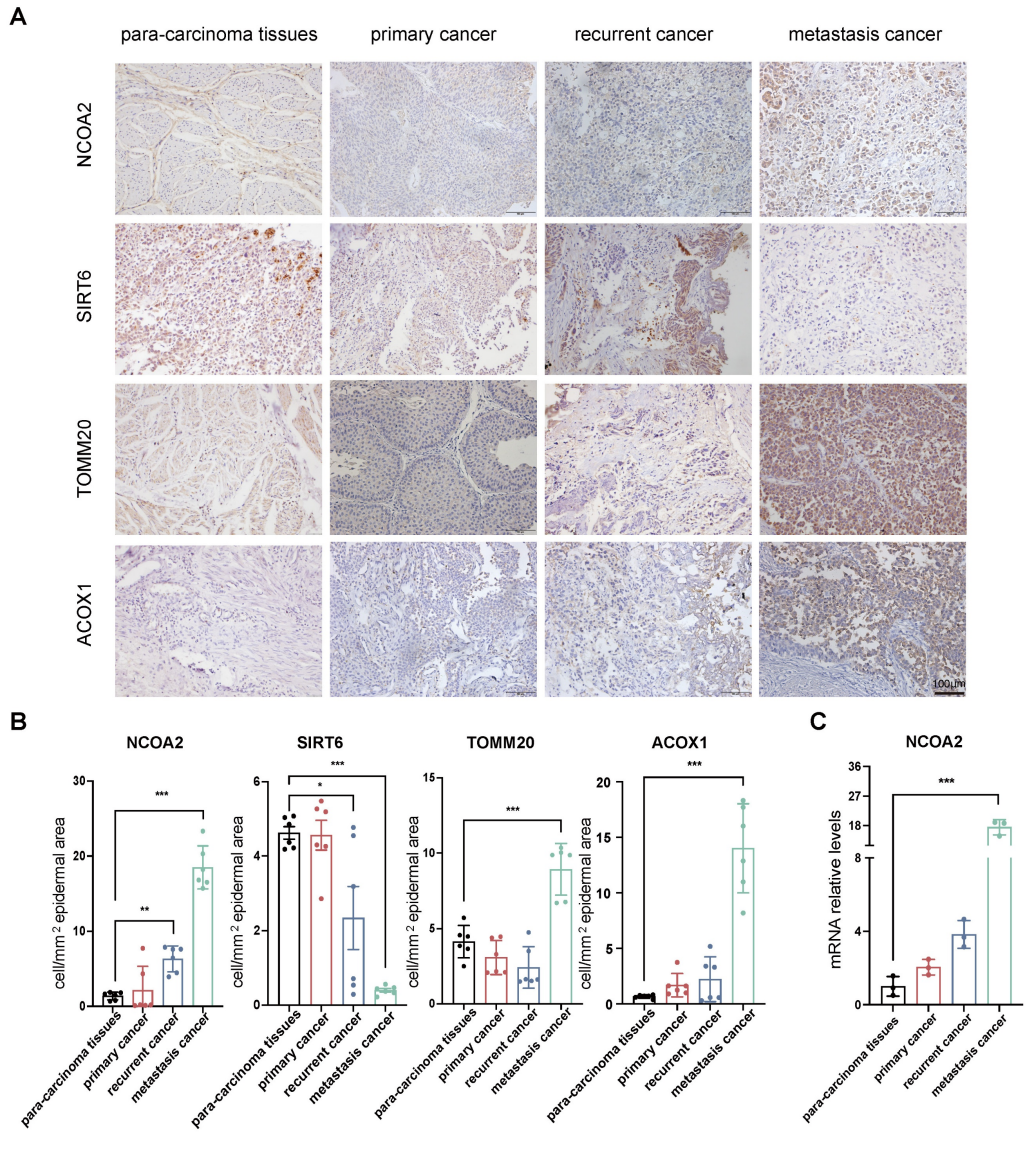
** Clinical relevance of NCOA2 acetylation and its role in tumor progression and metastasis in bladder cancer. (A-B)** Immunohistochemical (IHC) staining of NCOA2, SIRT6, TOMM20, and ACOX1 in tumor samples from patients with primary, recurrent, and metastatic bladder cancer, as well as matched adjacent normal tissues (n=6). **(A)** Representative IHC images. **(B)** Quantitative analysis of staining intensity. **(C)** Quantitative PCR (qRT-PCR) analysis of NCOA2 mRNA expression in tumor tissues from patients at different clinical stages compared with adjacent normal tissues (n=3). All quantitative data are presented as mean ± SD from at least three independent experiments. Statistical comparisons were performed using one-way ANOVA followed by Tukey's post hoc test. Data are presented as mean ± SD. *P* values are indicated as follows: * *p* < 0.05, ** p < 0.01, *** p < 0.001, **** *p* < 0.0001; ns = not significant, compared to the control group.^ ##^ *p < 0.01,* ^###^* p < 0.001*, ^####^ *p* < 0.0001; ns = not significant, compared to the vehicle group.

To determine the clinical relevance of the SIRT6 and NCOA2, we first analyzed their expression in bladder cancer samples, including primary, recurrent, and metastatic cancer. Compared with matched para-carcinoma tissues, tumors with lymphatic metastasis exhibited significantly elevated expression levels of NCOA2, translocase of outer mitochondrial membrane 20 (TOMM20), and ACOX1, while SIRT6 expression was markedly reduced, respectively (Fig. 7A and B). qRT-PCR analysis revealed consistent expression trends (Fig. 7C). These observations suggest that downregulation of SIRT6-resulting in impaired deacetylation of NCOA2-along with mitochondrial dysfunction and dysregulated fatty acid β-oxidation, may collectively promote the metastatic potential of bladder cancer cells.

## Discussion

In this study, we uncovered a novel regulatory axis involving SIRT6, NCOA2, and PPARα that mediates apigenin-induced mitochondrial dysfunction and metabolic reprogramming in bladder cancer cells. Despite advances in treatment, bladder cancer remains a major clinical challenge^41^, with high recurrence and progression rates, particularly in patients with muscle-invasive disease or those resistant to conventional therapies^42^-^44^. Natural compounds such as apigenin exhibit broad anticancer properties across multiple tumor types^45^-^47^. Previous studies have shown that apigenin can modulate diverse signaling cascades, including PI3K/Akt/mTOR, NF-κB, MAPK, and p53, leading to reduced tumor growth^48^, induction of apoptosis, and sensitization to chemotherapy or immunotherapy^49^-^51^. Beside canonical signaling cascades, the impact of apigenin on epigenetic and metabolic regulation remains poorly understood.

Our data revealed that NCOA2 deacetylation at K780/K785 significantly enhances PPARα activation. NCOA2 is a nuclear receptor coactivator whose activity is regulated by acetylation status^52^. Recent work has shown that SIRT6 can deacetylate NCOA2 at K780, enhancing its ability to activate PPARα signaling and drive lipid metabolic programs^38^. While SIRT6-PPARα signaling has been implicated in lipid metabolism and mitochondrial regulation in other biological contexts, particularly in metabolic tissues, its role in bladder cancer has not been previously characterized^47^, ^53^. Our study extends this existing knowledge by identifying a disease- and compound-specific SIRT6-NCOA2-PPARα regulatory mechanism engaged by apigenin in bladder cancer. Notably, the relationship between lipid metabolic activation and tumor fitness is highly context-dependent. While many tumors exploit fatty acid oxidation (FAO) to support energy production and metastatic traits, increasing evidence indicates that excessive or dysregulated lipid oxidation can instead be cytotoxic by inducing lipotoxic stress and mitochondrial dysfunction. Consistent with this concept, our data support that apigenin induces a maladaptive lipid metabolic state characterized by heightened PPARα-driven FAO, ROS accumulation, and mitochondrial depolarization, thereby converting metabolic activation into lethal mitochondrial stress^54^-^56^.

Cellular responses to flavonoids are highly context-dependent. While certain apigenin derivatives, such as quercetin or genistein, have been reported to alleviate cellular stress and stabilize mitochondrial membrane potential in specific experimental settings^57^, our findings demonstrate that apigenin induces mitochondrial dysfunction in bladder cancer cells. Mechanistically, amplified PPARα signaling imposes an excessive mitochondrial metabolic burden, leading to mitochondrial depolarization, ROS accumulation, and ATP depletion, which can be effectively reversed by pharmacological inhibition of PPARα using the selective antagonist NXT629. Time-course analyses further reveal a biphasic mitochondrial response, with early metabolic activation followed by progressive mitochondrial failure and apoptotic cell death upon sustained apigenin exposure.

Our findings further demonstrate that dual-site deacetylation of NCOA2 at K780 and K785 produces stronger PPARα activation. Given that SIRT6 regulates multiple non-histone substrates and is frequently downregulated in aggressive and metastatic bladder cancer, correlating with poor prognosis^58^, ^59^, enhancement of SIRT6-mediated deacetylation by apigenin—rather than indiscriminate enzymatic activation—may restore metabolic control and impose vulnerability in advanced disease.

Despite promising findings, our study is limited by the absence of *in vivo* genetic models and the lack of site-specific antibodies, which restricted validation of the SIRT6-NCOA2 axis and direct monitoring of endogenous NCOA2 acetylation at K780/K785. It should also be acknowledged that apigenin, like many natural compounds and clinically used anticancer agents, likely exerts its biological effects through polypharmacological mechanisms rather than a single molecular target. Notably, apigenin and several structurally related flavonoids, including quercetin and genistein, have been reported to act as pan-transporter inhibitors and have been explored for reversing multidrug resistance by attenuating transporter-mediated drug efflux, an aspect that may also be relevant in bladder cancer^60^. In this context, our findings define a dominant and mechanistically coherent metabolic pathway engaged by apigenin in bladder cancer, without excluding additional contributory targets, consistent with the emerging concept that therapeutic efficacy often arises from coordinated modulation of multiple cellular networks^61^. In addition, the bioavailability and pharmacokinetic properties of apigenin remain challenges for clinical translation. Future studies incorporating optimized drug-delivery strategies and larger clinical cohorts will be required to fully assess therapeutic feasibility and translational relevance.

In conclusion, our data reveal a SIRT6-NCOA2-PPARα metabolic checkpoint that is the base of apigenin-induced metabolic stress in bladder cancer. By promoting SIRT6-dependent NCOA2 deacetylation, apigenin is associated with fatty acid β-oxidation, ROS overproduction and mitochondrial failure. This discovery provides mechanistic insight into the anticancer activity of natural compounds and highlights a promising therapeutic target for bladder cancer.

## Supplementary Material

Supplementary figures and tables 1-3.

Supplementary table 4.

## Data Availability

The data that support the findings of this study are available from the corresponding author upon reasonable request and with the permission of the authors.

## Abbreviations

NCOA2: Nuclear Receptor Coactivator 2
ROS: Reactive oxygen species
VEGF-β: Vascular endothelial growth factor-β
TFs: Transcription factors
PPI: Protein-protein interaction
SDC: sodium deoxycholate
DTT: dithiothreitol
IAA: iodoacetamide
AS: Alternative splicing
ALT: Alanine aminotransferase
AST: Aspartate aminotransferase
BUN: Blood urea nitrogen
GLDH: Glutamate dehydrogenase
CETSA: Cellular thermal shift assay
IHC: Immunohistochemistry
PBS: Phosphate-buffered saline
qRT-PCR: Quantitative real-time polymerase chain reaction
cDNA: Complementary deoxyribonucleic acid
NAC: N-acetylcysteine
Co-IP: Co-immunoprecipitation
SD: Standard deviation
ANOVA: Analysis of variance
CDX: Cell-derived xenograft
PDX: Patient-derived xenograft
RRM: RNA recognition motif
GSEA: Gene set enrichment analysis
KEGG: Kyoto encyclopedia of genes and genomes
PPAR: Peroxisome proliferator-activated receptor
ACOX1: Peroxisomal acyl-coenzyme A oxidase 1
FASN: Fatty acid synthase
CPT1A: Carnitine palmitoyltransferase 1A
ACC: Acetyl-CoA carboxylase
MMP: Mitochondrial membrane potential
TOMM20: Translocase of outer mitochondrial membrane 20
GO: Gene ontology
BP: Biological process
NES: Normalized enrichment score
