# Supplementary material for: Apigenin Suppresses Bladder Cancer via the SIRT6-NCOA2-PPARα Axis
Source: Int J Biol Sci. 2026 Feb 26;22(6):3070–86. doi: 10.7150/ijbs.128177 (PMC13050455; doi:10.7150/ijbs.128177)
Supplement: Supplementary file 1 — Supplementary figures and tables 1-3. [file ijbsv22p3070s1.pdf]

Supplementary Figures

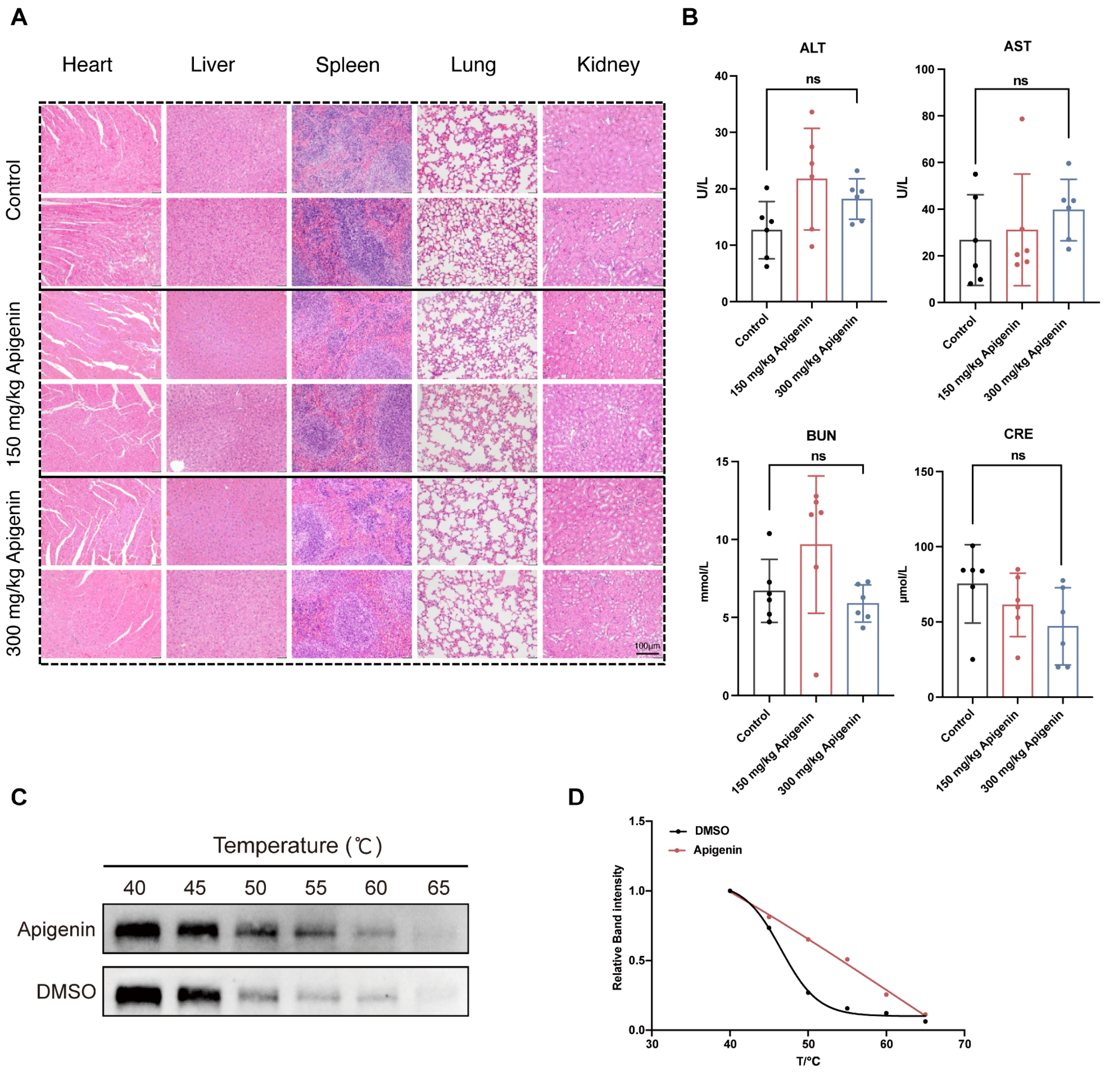

Figure S1. (A) Immunohistochemical (IHC) staining of peripheral organs in mice after oral administration of apigenin. **(B)** Serum levels of alanine aminotransferase (ALT), aspartate aminotransferase (AST), blood urea nitrogen (BUN), and creatinine (CER) were measured by ELISA (n = 6). **(C-D)** Thermal shift assay evaluating the binding of apigenin to SIRT6 protein. Control: DMSO. Left panel: SIRT6 protein bands; right panel: relative binding intensity (normalized to 40 °C). Statistical comparisons were performed using one-way ANOVA followed by Tukey's post hoc test. Data are presented as mean ± SD. *P* values are indicated as follows: * $p < 0.05$, ** $p < 0.01$, *** $p < 0.001$, **** $p < 0.0001$; ns = not significant, compared to the control group.

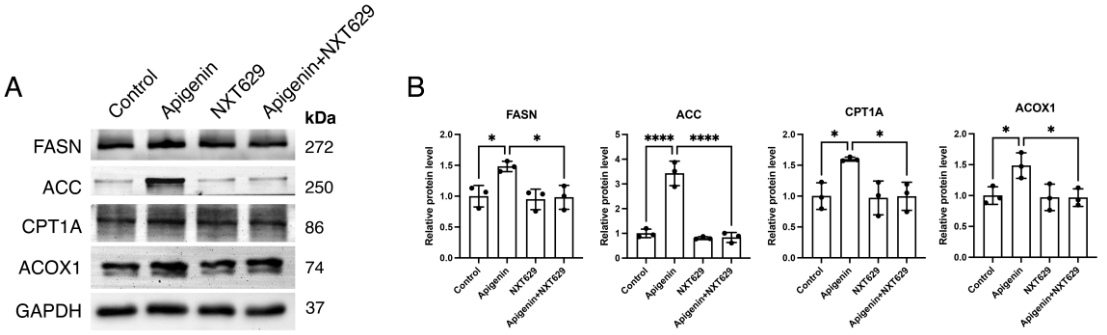

Figure S2. Apigenin alone or in combination with NXT629 regulates lipid metabolism-related enzymes in T24 cells. T24 cells were treated with apigenin (100 μM) alone or in combination with NXT629 for 24 h, followed by analysis of lipid metabolism-related enzyme expression. **(A)** Representative Western blot images showing the protein levels of FASN, ACC, CPT1A and ACOX1 after treatment with apigenin (100 μM) alone or together with NXT629 (5 μM). **(B)** Quantitative densitometric analysis of protein expression levels normalized to the corresponding loading control. All quantitative data are presented as mean ± SD from three independent experiments. Statistical comparisons were performed using one-way ANOVA followed by Tukey's post hoc test. *P* values are indicated as follows: * $p < 0.05$, **** $p < 0.0001$.

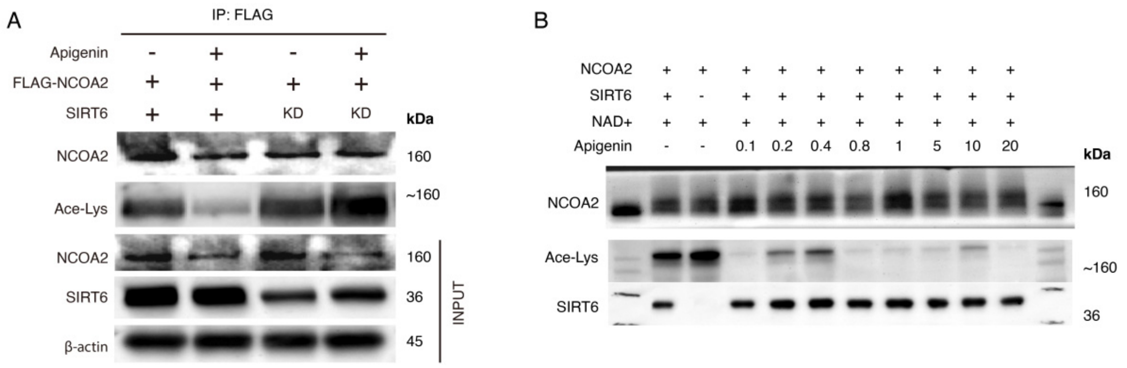

Figure S3. Apigenin promotes SIRT6-mediated deacetylation of NCOA2 in *vitro* and in *vivo*. **(A)** Knockdown of SIRT6 significantly reduced the deacetylation level of NCOA2 in cells. **(B)** In *vitro* deacetylation assays showed that apigenin at low concentrations (0.1-0.4 μM) inhibited SIRT6-mediated deacetylation of NCOA2, whereas higher concentrations (> 0.4 μM) enhance SIRT6-mediated NCOA2 deacetylation.

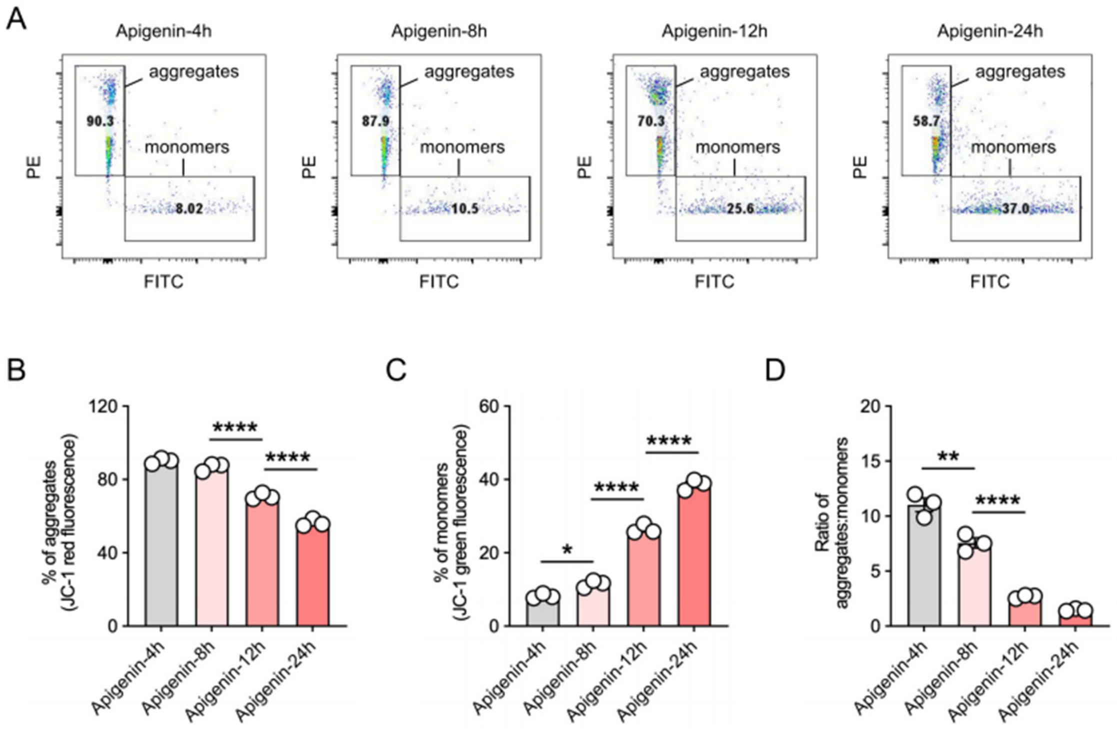

Figure S4. Apigenin alters mitochondrial membrane potential in T24 cells in a time-dependent manner. T24 cells were treated with apigenin (100 μM) for 4, 8, 12, or 24 h, and mitochondrial membrane potential was assessed using JC-1 staining. **(A)** Representative flow cytometry plots showing JC-1 monomers and aggregates at each time point. **(B)** Quantification of JC-1 aggregate levels. **(C)** Quantification of JC-1 monomer levels. **(D)** Ratio of JC-1 aggregates to monomers. All quantitative data are presented as mean ± SD from three independent experiments. Statistical comparisons were performed using one-way ANOVA followed by Tukey's post hoc test. *P* values are indicated as follows: * $p < 0.05$, ** $p < 0.01$, **** $p < 0.0001$.

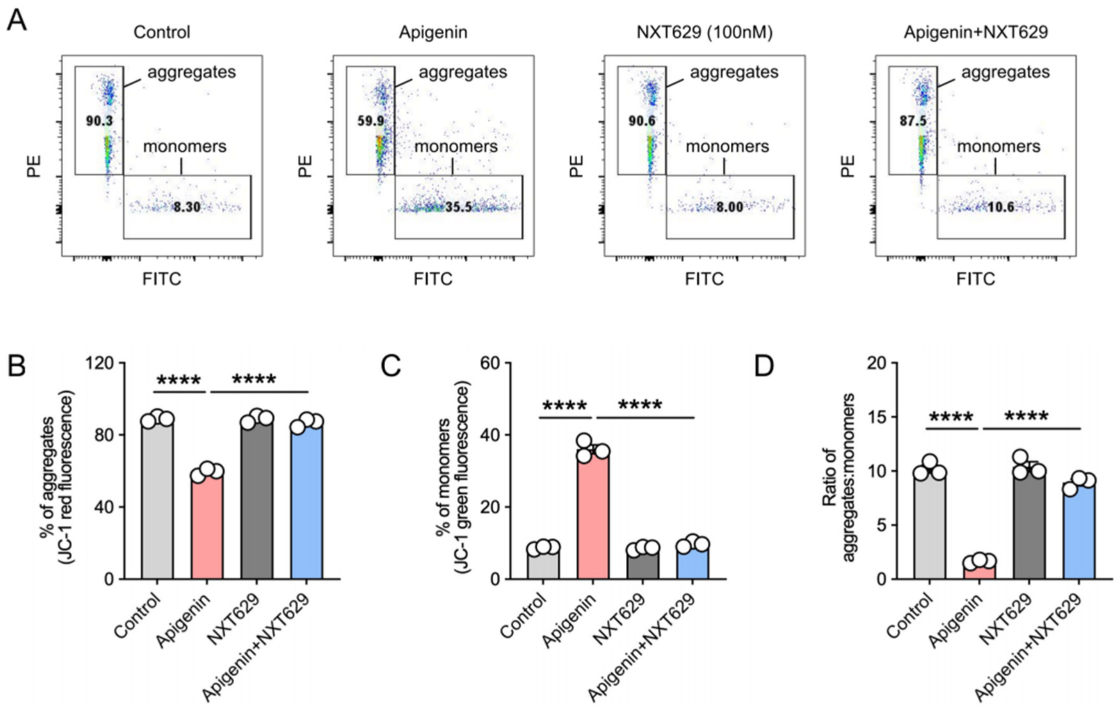

Figure S5. Combined treatment with apigenin and NXT629 affects mitochondrial membrane potential in T24 cells. T24 cells were treated with apigenin (100 μM) alone or in combination with NXT629 for 24 h, followed by JC-1 staining to assess mitochondrial membrane potential. **(A)** Representative flow cytometry plots of JC-1 monomers and aggregates. **(B)** Quantification of JC-1 aggregate levels. **(C)** Quantification of JC-1 monomer levels. **(D)** Ratio of JC-1 aggregates to monomers. All quantitative data are presented as mean ± SD from three independent experiments. Statistical comparisons were performed using one-way ANOVA followed by Tukey's post hoc test. *P* values are indicated as follows: **** $p < 0.0001$.

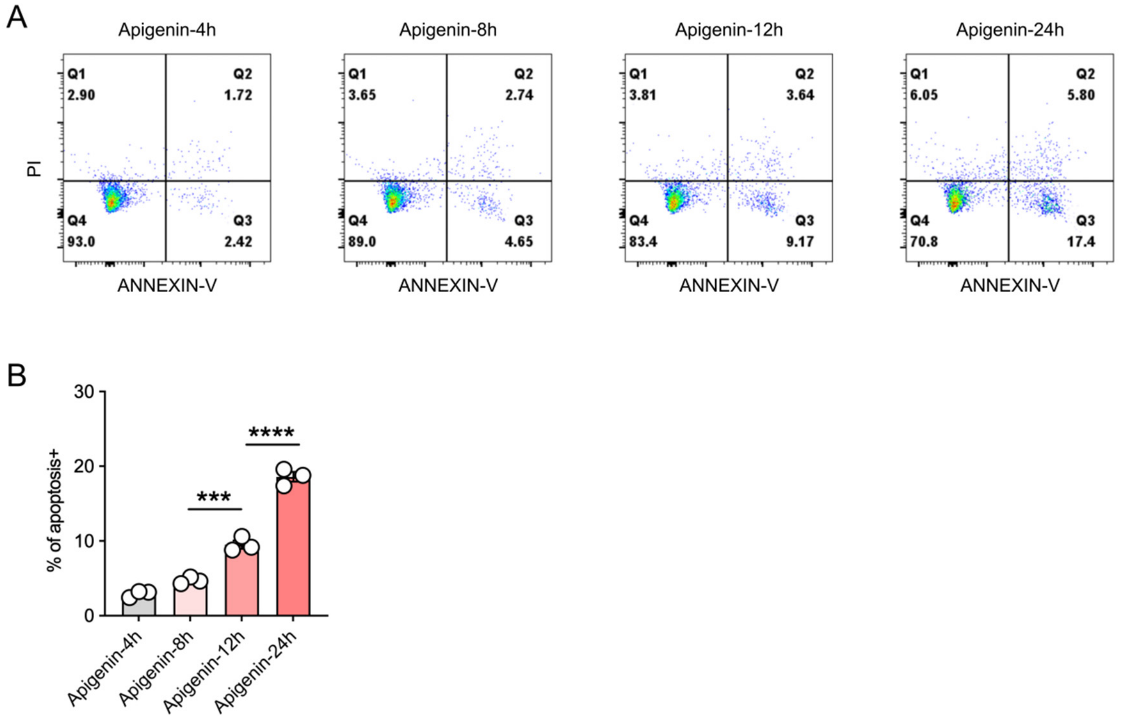

Figure S6. Time-dependent effects of apigenin on apoptosis and necrosis in T24 cells. T24 cells were treated with apigenin (100 μM) for 4, 8, 12, or 24 h, and cell apoptosis and necrosis were analyzed by flow cytometry. **(A)** Representative flow cytometry plots showing apoptotic and necrotic cells at each time point. **(B)** Statistical analysis of apoptosis rates. All quantitative data are presented as mean ± SD from three independent experiments. Statistical comparisons were performed using one-way ANOVA followed by Tukey's post hoc test. *P* values are indicated as follows: *** $p < 0.001$, **** $p < 0.0001$.

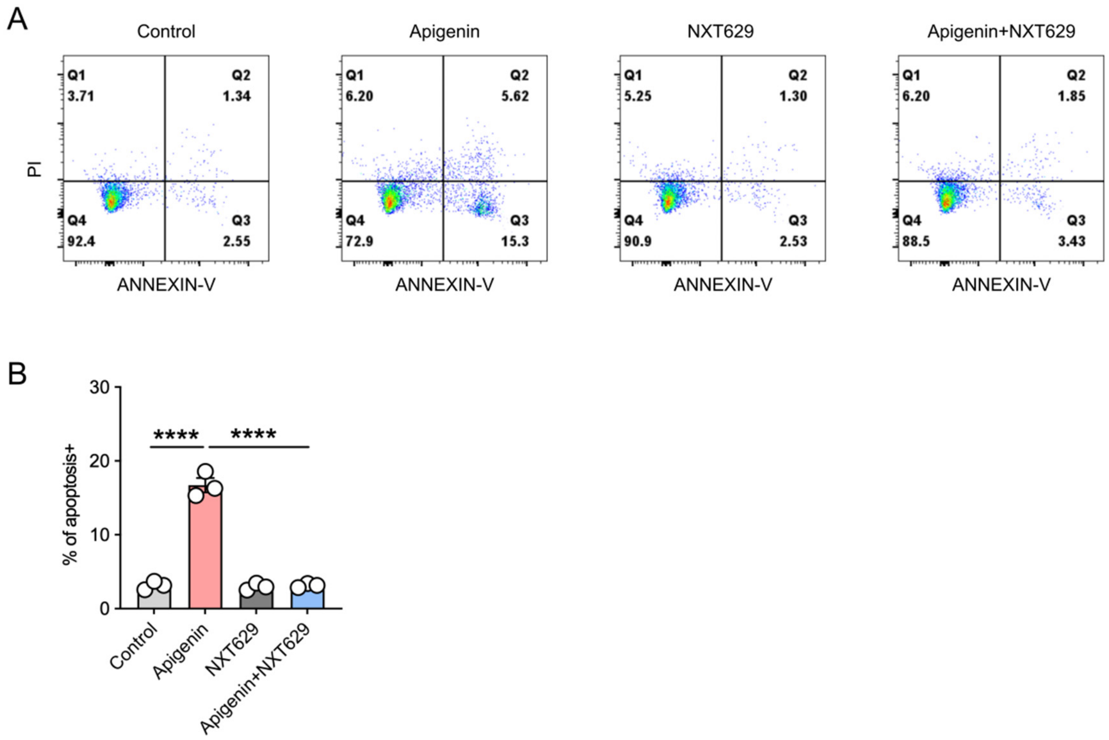

Figure S7. NXT629 attenuates apigenin-induced apoptosis in T24 cells. T24 cells were treated with apigenin (100 μM) alone or in combination with NXT629 for 24 h, and apoptosis and necrosis were assessed by flow cytometry. **(A)** Representative flow cytometry plots of apoptotic and necrotic cells. **(B)** Quantification of apoptosis rates. All quantitative data are presented as mean ± SD from three independent experiments. Statistical comparisons were performed using one-way ANOVA followed by Tukey’s post hoc test. *P* values are indicated as follows: **** $p < 0.0001$.

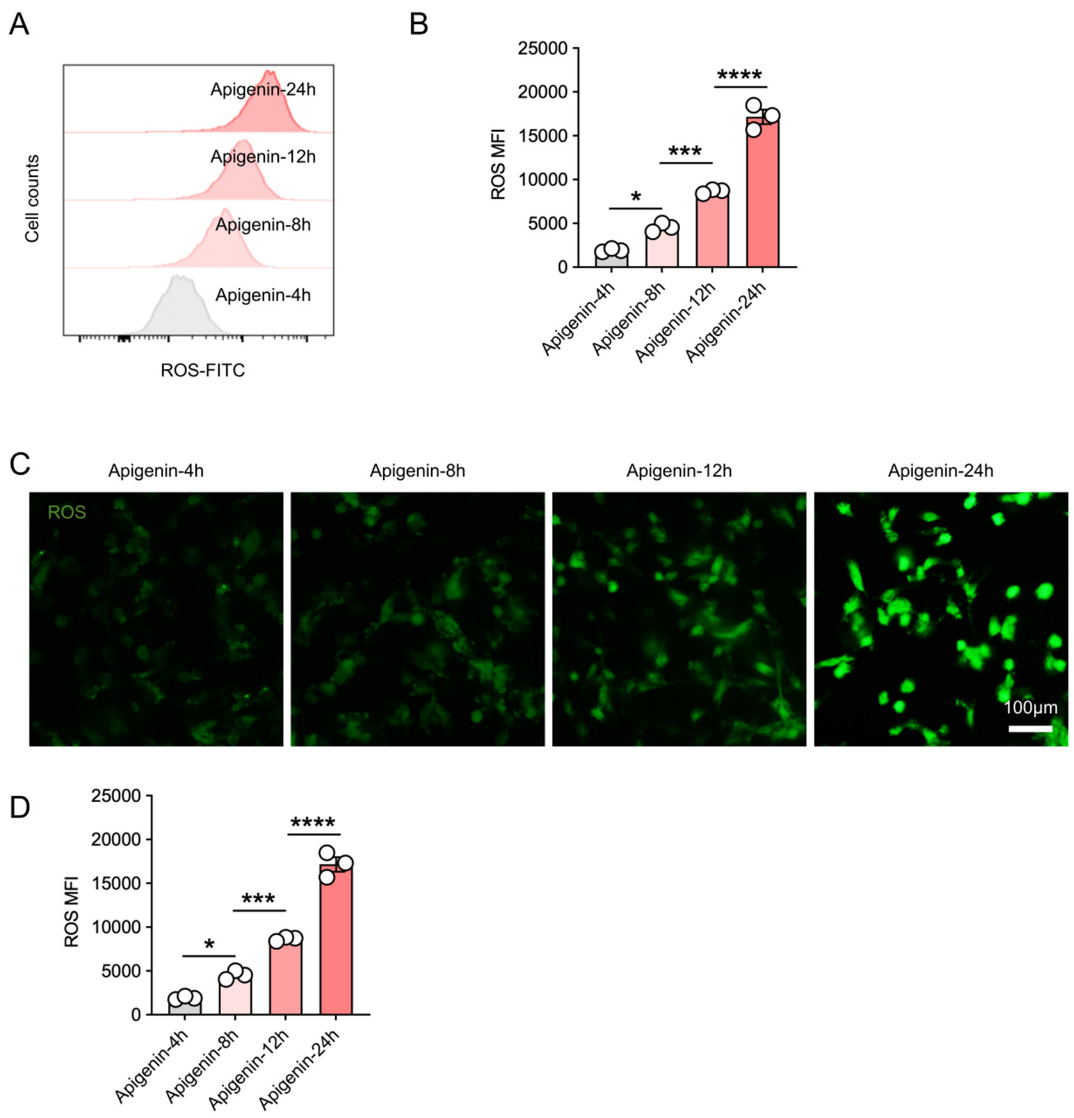

Figure S8. Apigenin increases intracellular ROS levels in T24 cells in a time-dependent manner. T24 cells were treated with apigenin (100 μM) for 4, 8, 12, or 24 h, and intracellular reactive oxygen species (ROS) levels were measured. **(A)** Representative flow cytometry plots of ROS levels at each time point. **(B)** Quantification of mean fluorescence intensity (MFI) of ROS measured by flow cytometry. **(C)** Representative fluorescence microscopy images showing ROS levels at each time point. **(D)** Quantification of average ROS fluorescence intensity observed by fluorescence microscopy. All quantitative data are presented as mean ± SD from three independent experiments. Statistical comparisons were performed using one-way ANOVA followed by Tukey's post hoc test. *P* values are indicated as follows: * $p < 0.05$, *** $p < 0.001$, **** $p < 0.0001$.

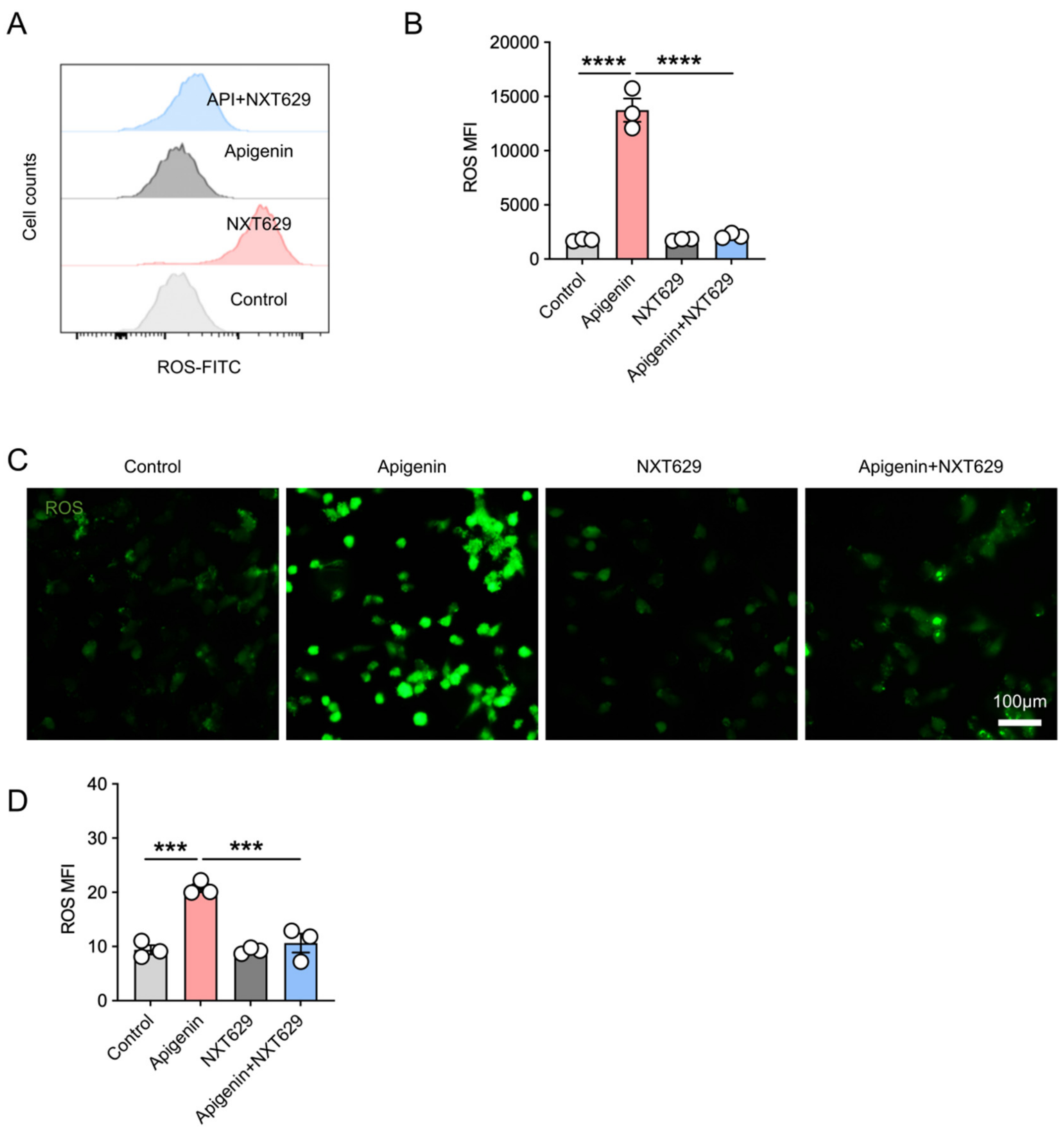

Figure S9. NXT629 reduces apigenin-induced intracellular ROS levels in T24 cells. T24 cells were treated with apigenin (100 μM) alone or in combination with NXT629 for 24 h, and intracellular ROS levels were detected. **(A)** Representative flow cytometry plots of ROS levels. **(B)** Quantification of ROS mean fluorescence intensity measured by flow cytometry. **(C)** Representative fluorescence microscopy images of intracellular ROS. **(D)** Quantification of average ROS fluorescence intensity observed by fluorescence microscopy. All quantitative data are presented as mean ± SD from three independent experiments. Statistical comparisons were performed using one-way ANOVA followed by Tukey's post hoc test. *P* values are indicated as follows: *** $p < 0.001$, **** $p < 0.0001$.

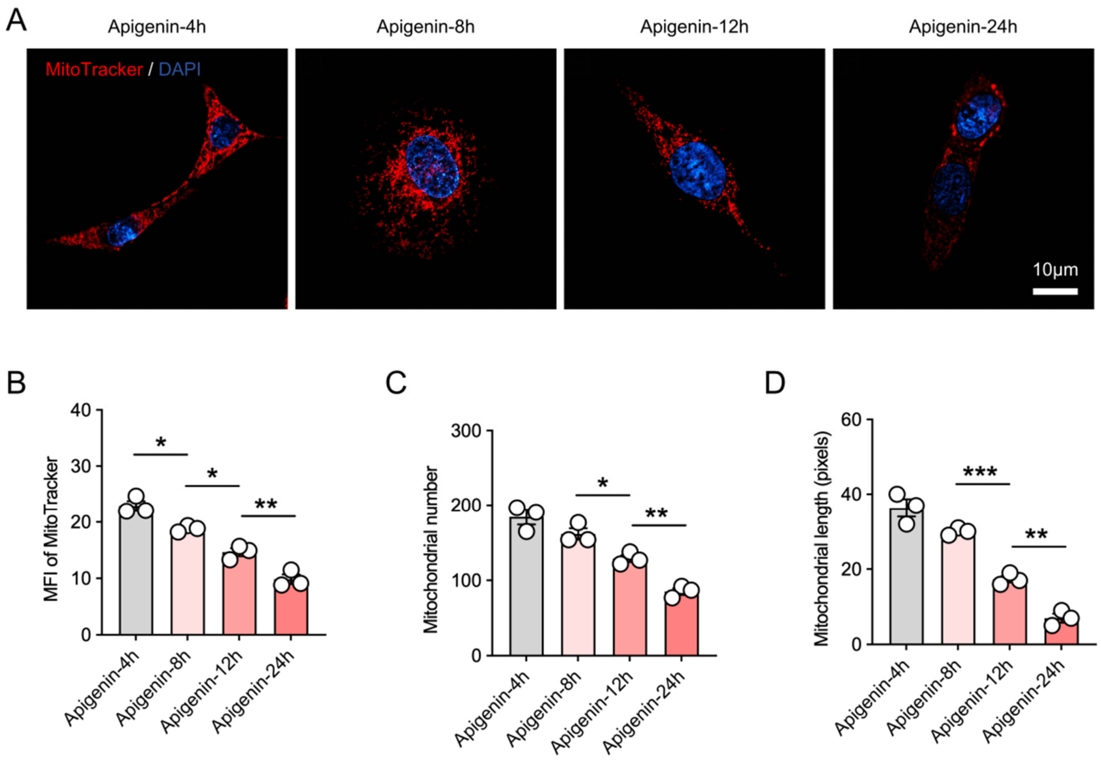

Figure S10. Apigenin regulates mitochondrial dynamics in T24 cells in a time-dependent manner. T24 cells were treated with apigenin (100 μM) for 4, 8, 12, or 24 h, and mitochondria were labeled using MitoTracker. **(A)** Representative images of MitoTracker-stained mitochondria. **(B)** Quantification of MitoTracker mean fluorescence intensity. **(C)** Quantification of mitochondrial number. **(D)** Quantification of mitochondrial length. All quantitative data are presented as mean ± SD from three independent experiments. Statistical comparisons were performed using one-way ANOVA followed by Tukey's post hoc test. *P* values are indicated as follows: * $p < 0.05$, ** $p < 0.01$, *** $p < 0.001$.

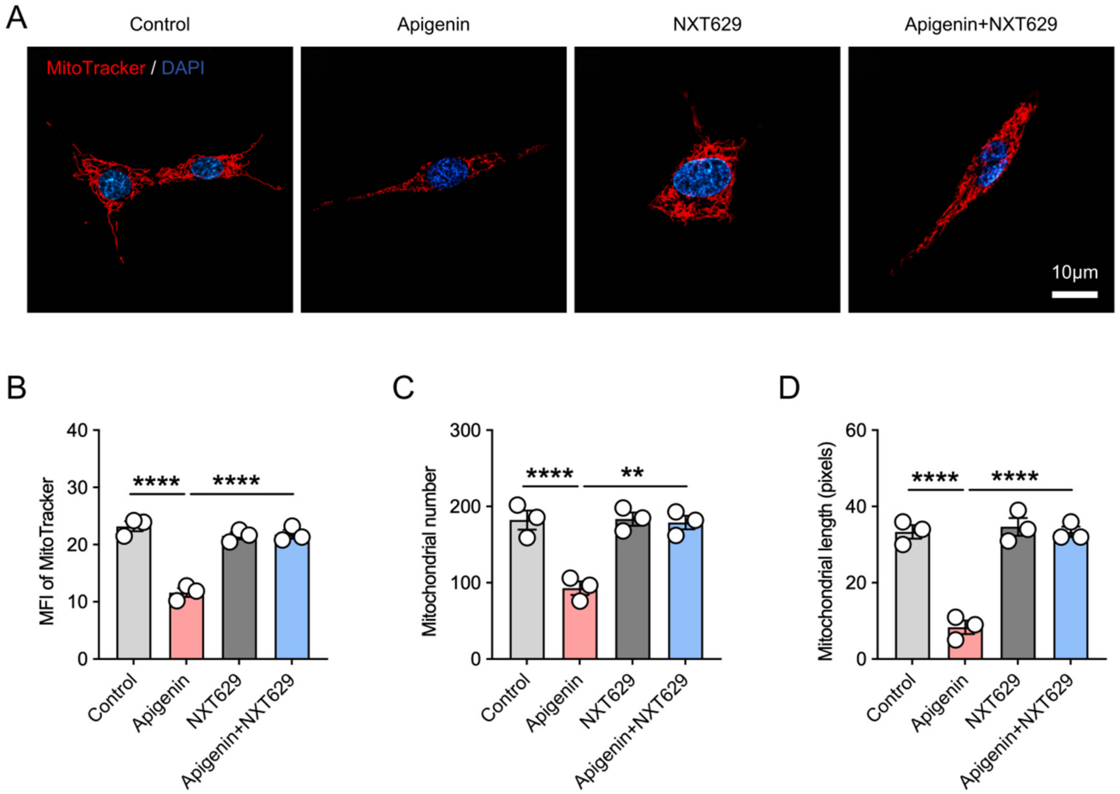

Figure S11.NXT629 alleviates apigenin-induced mitochondrial morphology abnormalities in T24 cells. T24 cells were treated with apigenin (100 μM) alone or in combination with NXT629 for 24 h, and mitochondria were visualized using MitoTracker staining. **(A)** Representative images of MitoTracker-stained mitochondria showing changes in morphology after treatment. **(B)** Quantification of MitoTracker mean fluorescence intensity. **(C)** Quantification of mitochondrial number. **(D)** Quantification of mitochondrial length, demonstrating that NXT629 significantly alleviated the mitochondrial morphology abnormalities induced by apigenin treatment. All quantitative data are presented as mean ± SD from three independent experiments. Statistical comparisons were performed using one-way ANOVA followed by Tukey's post hoc test. *P* values are indicated as follows: ** $p < 0.01$, **** $p < 0.0001$.

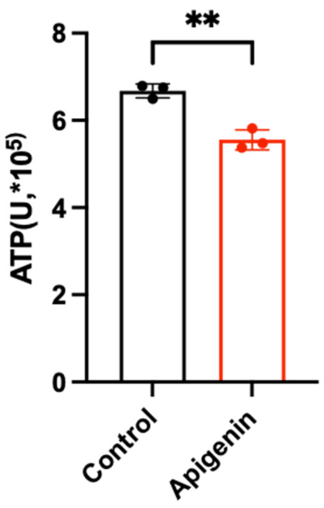

Figure S12. Apigenin reduces the ATP content in T24 cells. T24 cells were treated with apigenin (100 μM) for 24 hours, and the intracellular ATP level was measured. Statistical comparisons were performed using one-way ANOVA followed by Tukey's post hoc test. *P* values are indicated as follows: ** $p < 0.01$.

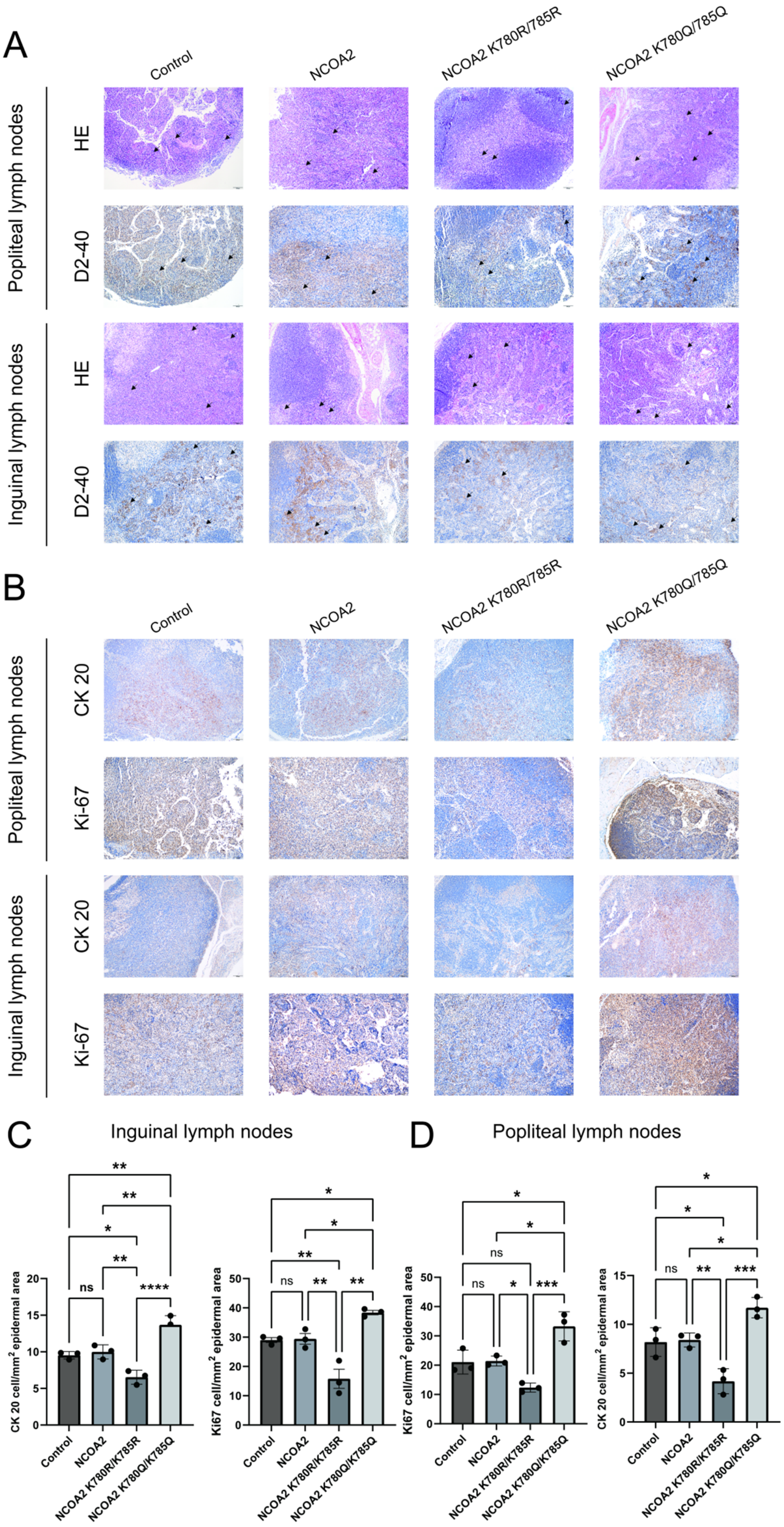
A
Control
NCOA2
NCOA2 K780R/785R
NCOA2 K780Q/785Q
Popliteal lymph nodes
Inguinal lymph nodes
HE
D2-40
B
CK 20
Ki-67
C
Inguinal lymph nodes
D
Popliteal lymph nodes
CK 20 cell/mm² epidermal area
Ki67 cell/mm² epidermal area
ns
NCOA2 K780R/K785R
NCOA2 K780Q/K785Q

Figure S13. HE and immunohistochemical staining of popliteal and inguinal lymph nodes in lymphatic metastasis model. **(A)** Schematic diagrams of HE staining and D2-40 IHC staining of popliteal and inguinal lymph nodes in the lymph node metastasis model, with arrows indicating the same position of lymphatic vessels; **(B)** Immunohistochemical staining showing the expression of CK 20 and Ki-67 in popliteal and inguinal lymph nodes of the lymph node metastasis model; The expression intensities of CK 20 and Ki-67 in popliteal lymph nodes **(C)** and inguinal lymph nodes **(D)** during quantitative immunohistochemical staining. Statistical comparisons were performed using one-way ANOVA followed by Tukey's post hoc test. *P* values are indicated as follows: * $p < 0.05$, ** $p < 0.01$, *** $p < 0.001$, **** $p < 0.0001$.

Supplementary Tables

Table S1. Distribution of Clinicopathological Characteristics Across Patients in qRT-PCR group

Characteristics	Total	para-carcinoma	Status		
			Primary	Recurrent	Metastatic
**Gender, n**					
Male	20	3	5	6	6
Female	4	3	1	0	0
Age, n					
＜65	8	4	0	0	4
≥65	16	2	6	6	2
**T stage, n**					
T1	12	1	6	5	0
T2	7	4	0	1	2
T3	5	1	0	0	4
T4	0	0	0	0	0
**N stage, n**					
N0	17	5	6	6	0
N1	0	0	0	0	0
N2	7	1	0	0	6
N3	0	0	0	0	0
**M stage, n**					
M0	24	6	6	6	6
M1	0	0	0	0	0
**Lymph Node Metastasis, n**					
Positive	7	1	0	0	6
Negative	17	5	6	6	0
**Stage, n**					
Stage I	12	1	6	5	0
Stage II	5	4	0	1	0
Stage III	3	1	0	0	2
Stage IV	4	0	0	0	4
**Pathological grade, n**					
high	17	6	0	5	6
low	7	0	6	1	0

Table S2. Distribution of Clinicopathological Characteristics Across Patients in the immunohistochemistry group

Characteristics	Total	para-carcinoma	Status		
			Primary	Recurrent	Metastatic
**Gender, n**					
Male	21	4	6	5	6
Female	3	2	0	1	0
**Age, n**					
＜65	7	4	0	1	2
≥65	17	2	6	5	4
**T stage, n**					
T1	12	3	5	4	0
T2	2	1	0	0	1
T3	10	2	1	2	5
T4	0	0	0	0	0
**N stage, n**					
N0	19	6	6	6	1
N1	3	0	0	0	3
N2	1	0	0	0	1
N3	1	0	0	0	1
**M stage, n**					
M0	24	6	6	6	6
M1	0	0	0	0	0
**Lymph Node Metastasis, n**					
Positive	5	0	0	0	5
Negative	19	6	6	6	1
**Stage, n**					
Stage I	12	3	5	4	0
Stage II	1	1	0	0	0
Stage III	11	2	1	2	6
Stage IV	0	0	0	0	0
**Pathological grade, n**					
high	22	6	5	5	6
low	2	0	1	1	0

Table S3. qRT-PCR primers employed in the present study.

Gene	Forward primer (5'-3')	Reverse primer (5'-3')
FASN	TTCTACGGCTCCACGCTCTTCC	GAAGAGTCTTCGTCAGCCAGGA
ACOX1	GGCGCATACATGAAGGAGACCT	AGGTGAAAGCCTTCAGTCCAGC
CPT1A	GATCCTGGACAATACCTCGGAG	CTCCACAGCATCAAGAGACTGC
ACC	TTCACTCCACCTTGTCAGCGGA	TTCACTCCACCTTGTCAGCGGA
NCOA2	TGGGGCCTATGATGCTTGAG	GGTTTTTGACAAATTCCGTGTGG
GAPDH	GTCTCCTCTGACTTCAACAGCG	ACCACCCTGTTGCTGTAGCCAA
